# Global burden of maternal disorders, 1990–2021: insights from the Global Burden of Disease Study 2021 and challenges for achieving 2030 Sustainable Development Goals

**DOI:** 10.7189/jogh.16.04147

**Published:** 2026-05-15

**Authors:** Junliang Zhu, Changyu Ni, Siying Wei, Jianing Bi, Xiaojin Wang, Jinxin Zheng, Xueying Xu, Wenwen Lv, Weijie Cai, Zhongxun Dong, Yejun Wu, Hongbo Liu, Bingshun Wang

**Affiliations:** 1 Department of Health Statistics, School of Public Health, China Medical University, Shenyang, China; 2 School of Public Health, Shanghai Jiao Tong University School of Medicine, Shanghai, China; 3 Institute of Clinical Medicine, Ruijin Hospital LuWan Branch, Shanghai Jiao Tong University School of Medicine, Shanghai, China; 4 Key Laboratory of Environmental Stress and Chronic Disease Control and Prevention, Ministry of Education, China Medical University, Shenyang, China; 5 School of Global Health, Chinese Center for Tropical Diseases Research, Shanghai Jiao Tong University School of Medicine, Shanghai, China; 6 One Health Center, Shanghai Jiao Tong University-The University of Edinburgh, Shanghai, China

## Abstract

**Background:**

The Sustainable Development Goals (SDGs) aim to reduce the global maternal mortality ratio (MMR) to below 70 deaths per 100 000 live births by 2030. However, progress has been uneven, with regional disparities and new challenges. We aimed to report global trends in maternal disorder burdens from 1990 to 2021.

**Methods:**

Using the Global Burden of Disease (GBD) 2021 database, we systematically analysed trends in incidence, mortality, and disability-adjusted life-years (DALYs) associated with maternal disorders from 1990 to 2021 and employed age-period-cohort (APC) models to assess changes in these indicators. For selected age-specific analyses, we additionally expressed incidence and DALY rates per 100 000 live births using age-specific fertility rates. We used the Bayesian APC model to forecast trends in maternal disorder incidence and mortality from 2022 to 2036.

**Results:**

From 1990 to 2021, there was a decrease in global incidence (38.5%), mortality (43.2%), and DALYs (61.4%) of maternal disorders. However, significant regional disparities persist. The burden was considerably lower in higher-sociodemographic-index regions than in sub-Saharan Africa. Maternal haemorrhage and hypertensive disorders continue to represent the primary causes of mortality, with human immunodeficiency virus (HIV)/acquired immunodeficiency syndrome (AIDS)-related deaths increasing, particularly among women aged 30–34 years and older. The APC model revealed that the age effect on maternal disorders initially decreased and then increased. The projected MMR of 136.5 (95% confidence interval = 100.2–172.7) per 100 000 live births by 2030 falls short of the SDG target of 70 maternal deaths per 100 000 live births, indicating a markedly insufficient annual reduction rate.

**Conclusions:**

Despite a decrease in the global burden of maternal disorders, regional disparities persist. Major challenges include haemorrhage, hypertensive disorders, and increasing HIV/AIDS-related deaths. Achieving SDG targets by 2030 requires universal access to quality maternal care.

In recent decades, there has been an increasing focus on addressing global maternal health concerns, which involves reducing maternal morbidity and mortality. This focus has been reflected in over 30 years of global initiatives, strategic plans, and quality enhancement activities [1–6]. It also serves as the main objective of numerous maternal health programmes at the regional, national, and local levels worldwide [7–9]. Maternal mortality is a crucial indicator of a nation's development and reflects the complex interactions among public health, social, and economic factors. The Millennium Development Goals aimed to reduce maternal mortality by 75% by 2015 [2], but many developing countries could not achieve this target [10]. Hence, in 2015, the Sustainable Development Goals (SDGs) established a new target to reduce the maternal mortality ratio (MMR) to 70 per 100 000 live births by 2030 [4]. However, achieving this goal remains difficult. According to the World Health Organization's latest report, the global MMR was 223 per 100 000 live births in 2020 [11]. Additionally, since 2016, progress in reducing MMR has stagnated in regions such as sub-Saharan Africa and South Asia. During the same period, MMR increased in Europe and North America [11]. This suggests that global progress in improving maternal health has stalled. Therefore, a comprehensive analysis of the changing trends in maternal disorder burden is necessary for identifying emerging challenges and providing insights for policymaking.

Although some studies have described global trends in maternal disorders, they often lack comprehensive analyses that cover recent periods and projections or focus primarily on specific regions or populations [12–17]. Moreover, health inequalities are increasingly acknowledged as significant contributors to maternal health outcomes [18,19], with disparities in access to care, socioeconomic status, and education contributing to the inequitable distribution of healthcare resources [20]. This is also reflected in the global changes in maternal mortality, with more than half of all maternal deaths occurring in fragile and humanitarian settings [21]. Therefore, understanding the spatial and temporal patterns of maternal disorder burden remains important for designing targeted interventions. Additionally, as global efforts intensify towards the SDGs, focusing solely on maternal mortality may be limited. The emphasis on maternal health is shifting beyond survival to overall health and well-being throughout the life course [22–24]. Consequently, incorporating more refined indicators, such as incidence and disability-adjusted life years (DALYs), is essential for gaining deeper insights into maternal quality of life and health outcomes.

Therefore, we used data from the Global Burden of Disease (GBD) 2021 to analyse the latest distribution and trends in the burden of maternal disorders across different global regions and to explore their relationship with socioeconomic development. Through descriptive analysis and age-period-cohort (APC) models, we aimed to assess the changes in the incidence, mortality, and DALY rates of maternal disorders from 1990 to 2021 and to project the disease burden from 2022 to 2036. Compared with previous GBD-based analyses, we integrated temporal decomposition with forward projections and evaluated the gap between projected trends and the SDG targets for maternal mortality. Our results could provide evidence for the development of effective maternal health interventions that contribute to reducing the global burden of maternal disorders.

## METHODS

We adhered to the Journal of Global Health’s Guidelines for Reporting Analyses of Big Data Repositories Open to the Public (Table S1 in the **Online Supplementary Document**) [25].

### Data sources

We sourced the data from the GBD 2021 database [26]. The GBD 2021 study assessed the burden of 371 diseases and injuries from 1990 to 2021 across 204 countries and territories [27]. The GBD estimates are generated through a comprehensive modelling framework that integrates multiple data sources and applies standardised statistical methods [27,28]. We used these GBD modelled estimates as standardised inputs for a secondary ecological analysis of temporal patterns and projections. We obtained the incidence, mortality, and DALY rates of maternal disorders, along with their 95% uncertainty intervals (UIs), by location (21 regions and 204 countries and territories), sex (female), age (10–54 years), year (1990–2021), and sociodemographic index (SDI) from the GBD 2021.

### Maternal disorders definition

We defined maternal disorders and their subcategories according to the GBD 2021 cause hierarchy and identified them based on International Classification of Diseases coding and GBD cause mapping [29]. Within the GBD framework, maternal causes are mutually exclusive and collectively exhaustive; therefore, each fatal or non-fatal event is assigned to a single underlying cause, avoiding double counting across categories. In this study, maternal disorders included overall maternal disorders, six nonfatal outcomes (*i.e.* abortion and miscarriage, ectopic pregnancy, maternal haemorrhage, maternal sepsis and other maternal infections, maternal hypertensive disorders, and maternal obstructed labour and uterine rupture), and four fatal outcomes (*i.e.* indirect maternal deaths, late maternal deaths, maternal deaths aggravated by human immunodeficiency virus (HIV)/acquired immunodeficiency syndrome (AIDS), and other direct maternal disorders).

### SDI definition

The SDI measures a country's development on a scale of 0–1 based on *per capita* income, average educational attainment, and total fertility rates under the age of 25 [30]. The GBD research divided 204 countries and regions into five SDI groups according to quintiles: high, high-middle, middle, low-middle, and low SDIs [31].

### Statistical analysis

We grouped maternal ages into nine categories (*i.e.* 10–14, 15–19, 20–24, 25–29, 30–34, 35–39, 40–44, 45–49, and 50–54 years). Age-specific incidence and DALY rates based on the female population denominator may be influenced by variations in fertility patterns. To better approximate maternal risk conditional on pregnancy exposure, we used age-specific fertility rate (ASFR) data from GBD 2021 to rescale selected age-specific incidence and DALY rates. Specifically, we divided the original rates by the corresponding ASFR values and expressed them per 100 000 live births. We obtained age-specific mortality data from the MMR reported in GBD 2021. We obtained UIs for the transformed indicators by applying the same ASFR-based rescaling procedure to the published GBD point estimates and corresponding upper and lower bounds. We did not introduce additional variance modelling during this process.

We assessed the association between maternal disorder burden and SDI for the 21 GBD regions from 1990 to 2021. We used locally weighted scatterplot smoothing to visualise the overall SDI-burden gradient. We interpreted the regions located above the smoothed curve as having a higher burden, and those located below as having a lower burden than the overall development gradient.

We used an APC model to estimate the independent effects of age, period, and birth cohort on the incidence, mortality, and DALY rates of maternal disorders. We divided maternal age into nine groups (from 10–14 to 50–54), and six consecutive five-year periods (1992–1996 to 2017–2021), generating corresponding five-year birth cohorts (1942–2007). The APC model enables the decomposition of temporal variations into age, period, and cohort components. Because age, period, and cohort are linearly dependent (cohort = period – age), APC models are subject to the well-known identifiability problem. To address this issue, we applied a constraint-based parameterisation in which we defined reference categories for period and cohort effects. We selected period 2002–2006 and the birth cohort of 1972 as reference categories because they lie near the temporal midpoint of the observation window, a common strategy used to reduce boundary effects and improve interpretability. Under this parameterisation, the estimated age, period, and cohort effects represent deviations relative to the selected reference groups rather than uniquely identifiable linear components. We used age-specific live birth data from the GBD 2021 as the reference population.

To project future trends, we used a Bayesian APC (BAPC) model to forecast the global incidence and mortality of maternal disorders from 2022 to 2036. In this model, age, period, and cohort effects were assigned second-order random walk priors, assuming that temporally adjacent groups have similar effects and allowing smooth changes over time. We estimated the posterior distributions using the integrated nested Laplace approximation, which provides efficient Bayesian inference without relying on Markov chain Monte Carlo sampling [32,33]. We obtained population projections from the Institute for Health Metrics and Evaluation global population forecast data set [34,35]. Standardisation followed the GBD 2021 standard population [36]. Because MMRs are expressed per 100 000 live births, we multiplied projected population counts for each age group from 2022 to 2036 by the ASFR observed in 2021 to estimate projected live births for each age group. We then used these projected live births for population normalisation, with the 2021 live births by age group serving as the reference standard.

We used the APC Web Tool [37] and *R*, version 4.2.1 (R Core Team, Vienna, Austria) for data processing.

## RESULTS

### Global maternal disorders incidence, mortality and DALYs

In 2021, there were 111.5 million cases of maternal disorders worldwide, with an age-standardised incidence rate (ASIR) of 4471.2 per 100 000 population, a 38.5% decrease since 1990 (**Table 1**). Maternal disorders led to 191.2 thousand deaths, with an MMR of 147.8 per 100 000 live births, down by 43.2%. The global DALYs for maternal disorders were 12.3 million, with an age-standardised DALY rate (ASDR) of 492.9 per 100 000 population, marking a 61.4% reduction since 1990.

**Table 1:** Incidence, deaths, and DALYs for maternal disorders in 2021 by SDI and GBD region*

	Incidence (95% UI)			Deaths (95% UI)			DALYs (95% UI)		
	**N in millions**	**ASIR per 100 000 population**	**% change in ASIR**	**N in thousands**	**MMR per 100 000 live births**	**% change in MMR**	**N in thousands**	**ASDR per 100 000 population**	**% change in ASDR**
**Overall**	111.5 (98.5, 125.2)	4471.2 (3949.3, 5018.7)	−38.5	191.2 (161.5, 227.1)	147.8 (125.3, 174.5)	−43.2	12 297.7 (10 567.7, 14511.5)	492.9 (423.6, 581.7)	−61.4
**Disease type**									
Maternal haemorrhage	14 (10.9, 17.7)	559.6 (436.8, 711.5)	−32.7	46.9 (39.1, 56.6)	36.2 (30.1, 43.6)	−58.2	2955.5 (2492.5, 3536.9)	118.5 (99.9, 141.8)	−71.6
Maternal sepsis and other maternal infections	19 (14.6, 24.1)	763.5 (585.6, 965.5)	−42.1	17.7 (14.6, 21.2)	13.7 (11.3, 16.4)	−30.0	1144.2 (957.0, 1352.0)	45.9 (38.4, 54.2)	−53.5
Maternal hypertensive disorders	18.1 (15.4, 21.5)	723.5 (615.5, 862.6)	−21.3	38.1 (31.9, 46.1)	29.5 (24.7, 35.4)	−27.9	2469.6 (2083.4, 2958.2)	99.0 (83.5, 118.6)	−51.5
Maternal obstructed labour and uterine rupture	13.5 (8.9, 19.0)	540.0 (358.3, 761.9)	−42.7	11.7 (9.8, 13.9)	9.0 (7.5, 10.8)	−47.5	1050.9 (883.6, 1256.1)	42.1 (35.4, 50.4)	−59.6
Maternal abortion and miscarriage	38.6 (29.9, 48.6)	1548.9 (1200.4, 1947.2)	−43.9	16.7 (14.1, 20.3)	12.9 (10.8, 15.6)	−64.2	1007.8 (855.3, 1215.0)	40.4 (34.3, 48.7)	−75.7
Ectopic pregnancy	8.4 (6.7, 10.4)	335.8 (266.6, 417.8)	−33.1	6.4 (5.4, 7.8)	5.0 (4.2, 5.9)	41.5	396.9 (332.6, 479.3)	15.9 (13.3, 19.2)	−5.9
Indirect maternal deaths				19.2 (16.4, 22.4)	14.8 (12.7, 17.3)	−41.6	1137.1 (971.5, 1329.7)	45.6 (38.9, 53.3)	−62.3
Late maternal deaths				5.6 (4.4, 6.9)	4.3 (3.4, 5.4)	−6.3	332.5 (263.6, 411.6)	13.3 (10.6, 16.5)	−38.8
Maternal deaths aggravated by HIV/AIDS				1.3 (0.8, 1.8)	1.0 (0.6, 1.4)	65.9	71.2 (44.0, 99.1)	2.9 (1.8, 4.0)	2.9
Other direct maternal disorders				27.6 (23.0, 33.9)	21.3 (17.8, 26.2)	−16.6	1731.9 (1456.2, 2076.7)	69.4 (58.4, 83.2)	−44.4
**SDI region**									
High SDI	8.1 (7.3, 9.0)	2629.3 (2355.4, 2899.7)	−38.6	1.8 (1.6, 2.1)	17.8 (15.2, 21.1)	3.6	140.8 (119.5, 168.5)	45.6 (38.7, 54.5)	−25.9
High-middle SDI	12.3 (11.0, 13.7)	3145.5 (2800.6, 3499.9)	−41.4	2.6 (2.2, 3.1)	22.1 (18.5, 26.8)	−64.4	200.0 (168.2, 237.6)	51.2 (43.0, 60.8)	−76.2
Middle SDI	28.0 (24.6, 31.6)	3544.8 (3116.7, 3996.7)	−49.0	23.1 (20.2, 26.8)	72.5 (63.1, 83.9)	−53.7	1531.1 (1355.8, 1758.4)	193.8 (171.6, 222.6)	−73.6
Low-middle SDI	32.2 (28.0, 36.8)	5005.7 (4351.8, 5733.5)	−47.8	59.7 (50.3, 71.6)	151.9 (129.1, 180.7)	−61.9	3870.0 (3307.4, 4569.8)	602.3 (514.8, 711.3)	−77.6
Low SDI	30.9 (27.4, 34.8)	8572.3 (7603.0, 9657.2)	−34.3	103.8 (85.4, 127.0)	286.5 (233.7, 349.9)	−44.6	6545.5 (5440.1, 7906.9)	1817.6 (1510.7, 2195.6)	−61.6
**GBD region**									
High-income Asia Pacific	0.7 (0.6, 0.8)	1387.5 (1221.3, 1591.2)	−65.7	0.1 (0.1, 0.1)	6.1 (5.2, 7.2)	−65.2	7.0 (5.7, 8.5)	14.2 (11.6, 17.4)	−68.6
High-income North America	3.1 (2.8, 3.4)	2917.7 (2640.6, 3209.2)	−36.7	1.1 (1.0, 1.3)	27.7 (23.9, 32.9)	125.1	80.4 (69.6, 94.1)	74.9 (64.9, 87.8)	29.6
Western Europe	3.6 (3.2, 4.1)	3006.5 (2659.2, 3361.7)	−16.9	0.2 (0.1, 0.2)	3.8 (3.4, 4.3)	−59.7	23.9 (17.7, 30.6)	19.8 (14.7, 25.4)	−43.8
Australasia	0.3 (0.3, 0.4)	3419.4 (2980, 3869.2)	−20.4	0.0 (0.0, 0.0)	3.4 (2.8, 4.1)	−51.7	2.1 (1.6, 2.8)	22.9 (16.9, 30.8)	−40.6
Southern Latin America	0.9 (0.8, 1.0)	4257.8 (3781.0, 4679.9)	−30.7	0.3 (0.2, 0.4)	38.9 (30.8, 49.0)	−32.4	22.4 (18.2, 27.1)	102.5 (83.4, 124.1)	−59.3
Andean Latin America	1.6 (1.4, 1.8)	7468.7 (6606.6, 8366.6)	−41.9	1.4 (1.0, 1.8)	110.3 (82.5, 143.9)	−48.8	85.5 (65.7, 110.9)	390.5 (300.3, 506.9)	−69.3
Tropical Latin America	3.4 (2.9, 3.9)	4442.3 (3850.4, 5132.3)	−40.6	2.1 (1.9, 2.3)	61.0 (55.6, 67.0)	−56.1	139.1 (126.6, 152.6)	184.4 (167.8, 202.3)	−67.1
Central Latin America	5.3 (4.7, 6.0)	6182.1 (5425.7, 7003.9)	−44.9	2.7 (2.3, 3.2)	69.6 (57.3, 83.0)	−30.2	180.9 (155.3, 214.0)	210.1 (180.4, 248.5)	−65.0
Caribbean	0.8 (0.7, 0.9)	5202.3 (4480.6, 5978.9)	−42.7	1.9 (1.3, 2.6)	235.1 (157.8, 326.0)	31.7	113.9 (77.9, 158.2)	744.7 (509.1, 1034.2)	−12.9
Eastern Europe	3.4 (3.0, 3.9)	5632.2 (4885.4, 6399.9)	−13.8	0.2 (0.2, 0.2)	11.2 (9.3, 13.6)	−75.5	24.7 (18.9, 31.7)	40.3 (30.9, 51.7)	−70.6
Central Europe	0.7 (0.6, 0.8)	2120.4 (1903.4, 2367.7)	−35.1	0.1 (0.0, 0.1)	5.1 (4.3, 6.0)	−82.7	6.6 (5.1, 8.3)	20.2 (15.5, 25.4)	−77.8
Central Asia	1.2 (1.1, 1.4)	4033.3 (3471.7, 4685.0)	−34.0	0.5 (0.4, 0.6)	22.6 (18.6, 27.0)	−54.8	32.9 (27.5, 38.7)	106.8 (89.5, 125.8)	−63.8
North Africa and Middle East	8.4 (7.3, 9.6)	4184.2 (3643.8, 4751.3)	−51.3	11.4 (8.3, 15.1)	94.0 (68.5, 126.0)	−48.9	730.7 (549.4, 950.9)	362.3 (272.4, 471.6)	−71.1
South Asia	28.1 (23.7, 32.7)	4521.1 (3820.0, 5263.1)	−53.1	46.2 (37.7, 56.5)	144.1 (118.2, 179.4)	−70.2	3093.3 (2575.8, 3737.8)	497.8 (414.5, 601.5)	−83.7
Southeast Asia	7.7 (6.9, 8.8)	3342.2 (2958.1, 3788.8)	−43.6	11.7 (9.5, 14.6)	104.6 (85.6, 130.2)	−59.1	718.1 (591.5, 887.0)	309.7 (255.1, 382.6)	−75.1
East Asia	9.9 (8.7, 11.1)	2277.0 (2001.5, 2567.3)	−59.0	1.7 (1.2, 2.3)	15.2 (10.8, 20.7)	−77.5	136.6 (104.6, 177.4)	31.5 (24.1, 40.9)	−87.9
Oceania	0.2 (0.2, 0.3)	5470.4 (4787.9, 6239.9)	−24.0	0.9 (0.6, 1.2)	203.7 (138.4, 278.6)	−12.4	54.6 (37.1, 76.0)	1233.1 (838.2, 1716.7)	−22.7
Western sub-Saharan Africa	13.7 (12.3, 15.3)	8639.2 (7766.5, 9601.3)	−27.7	53.6 (42.0, 68.5)	300.2 (235.9, 388.5)	−30.0	3376.7 (2690.5, 4268.7)	2125.5 (1693.6, 2687.0)	−48.5
Eastern sub-Saharan Africa	13.5 (11.9, 15.2)	9645.2 (8500.8, 10885.2)	−36.5	36.8 (29.8, 45.7)	267.0 (217.0, 329.0)	−45.7	2338.4 (1905.7, 2841.1)	1670.1 (1361.1, 2029.1)	−64.7
Central sub-Saharan Africa	3.1 (2.8, 3.5)	7156.9 (6396.5, 8053.4)	−40.3	15.5 (10.7, 21.4)	347.2 (244.2, 473.5)	−20.1	937.7 (659.1, 1276.5)	2158.5 (1517.2, 2938.4)	−48.4
Southern sub-Saharan Africa	1.6 (1.4, 1.9)	5936.0 (5116.2, 6860.3)	−22.7	3.0 (2.3, 4.0)	186.2 (144.5, 239.8)	−11.2	192.3 (150.5, 246.3)	702.1 (549.4, 899.1)	−44.7

ASIR – age-standardised incidence rate, ASDR – age-standardised DALY rate, DALYs – disability-adjusted life years, GBD – global burden of disease, MMR – maternal mortality ratio, SDI – sociodemographic index, UI – uncertainty intervals

*Percentage change for each metric is in comparison to corresponding point estimate in 1990.

The highest ASIR of maternal disorder regionally in 2021 was observed in the low-SDI group (ASIR = 8572.3), while the lowest was observed in the high-SDI group (ASIR = 2629.3). Similarly, the highest MMR was in the low-SDI group (MMR = 286.5) and the lowest was in the high-SDI group (MMR = 17.8). For DALYs, the low-SDI group had the highest rate (ASDR = 1817.6), while the high-SDI group had the lowest (ASDR = 45.6). Compared to the data from 1990, the percentage change in the ASIR in 2021 exhibited significant variation across the SDI regions. The middle-SDI region saw the largest decrease (−49.0%), while the low-SDI region had the smallest decrease (−34.3%). During the same period, the MMR declined the most in the high-middle SDI region (−64.4%) but slightly increased in the high-SDI region (3.6%). The ASDR decreased the most in the low-middle SDI region (−77.6%), with the smallest decrease in the high-SDI region (−25.9%).

Among the 21 regions, Eastern sub-Saharan Africa (ASIR = 9645.2), Western sub-Saharan Africa (ASIR = 8639.2), and Andean Latin America (ASIR = 7468.7) had the highest ASIR of maternal disorders in 2021, while high-income Asia Pacific (ASIR = 1387.5), Central Europe (ASIR = 2120.4), and East Asia (ASIR = 2277.0) had the lowest. Central sub-Saharan Africa (MMR = 347.2), Western sub-Saharan Africa (MMR = 300.2), and Eastern sub-Saharan Africa (MMR = 267.0) had the highest MMR, with the lowest rates in Australasia (MMR = 3.4), Western Europe (MMR = 3.8), and Central Europe (MMR = 5.1). Central sub-Saharan Africa (ASDR = 2158.5), Western sub-Saharan Africa (ASDR = 2125.5), and Eastern sub-Saharan Africa (ASDR = 1670.1) had the highest ASDR, while high-income Asia-Pacific (ASDR = 14.2), Western Europe (ASDR = 19.8), and Central Europe (ASDR = 20.2) had the lowest.

In 2021, ASIR of maternal disorders declined in all regions compared to 1990. The most significant declines were observed in high-income Asia Pacific (−65.7%), East Asia (−59.0%), and South Asia (−53.1%). In the same period, most regions showed a decrease in MMR, with the largest decreases in Central Europe (−82.7%), East Asia (−77.5%), and Eastern Europe (−75.5%). However, the MMR had increased in high-income North America (125.1%) and the Caribbean (31.7%). Similarly, most regions showed a decrease in ASDR, with the largest decreases in East Asia (−87.9%), South Asia (−83.7%), and Central Europe (−77.8%). High-income North America (29.6%) was the only region with an increase.

In 2021, the ASIR of various maternal disorders indicated lower rates in high-income regions and higher rates in middle- and low-income regions. The ASIR of maternal abortion and miscarriage was among the highest across all regions. However, maternal hypertensive disorders had significantly higher ASIR in sub-Saharan Africa. sub-Saharan Africa and Oceania also had markedly higher MMRs and DALYs than other regions. Maternal haemorrhage, hypertensive disorders, and other direct maternal disorders were the leading causes of maternal death in these regions (Figure S1, Panels A–C in the **Online Supplementary Document**).

**Figure 1:**
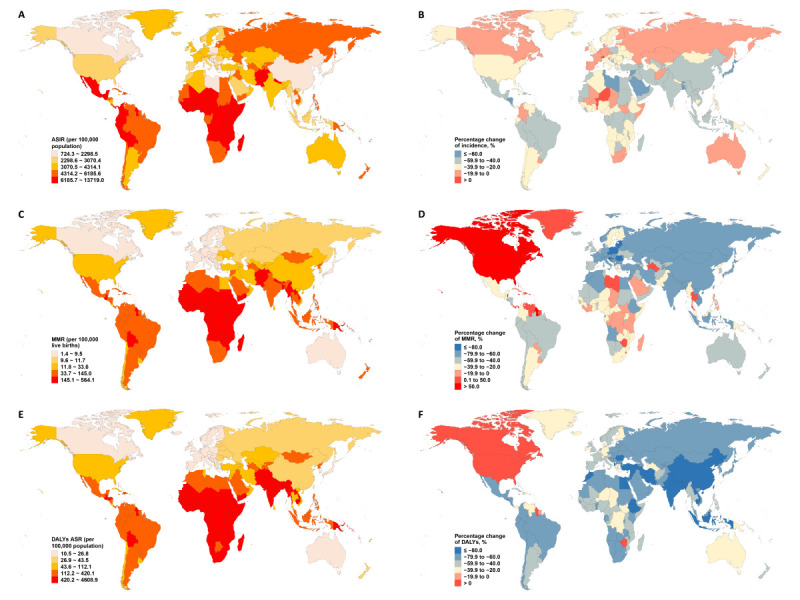
ASRs of incidence, mortality, and DALYs of maternal disorders in 204 countries and territories in 2021. **Panel A.** ASIR. **Panel B.** Percentage change of incidence. **Panel C.** MMR. **Panel D.** Percentage change of MMR. **Panel E.** DALYs ASR. **Panel F.** Percentage change of DALYs. The percentage change for each metric is in comparison to the rates in 1990. ASIR – age-standardised incidence rate, ASR – age-standardised rate, DALY – disability-adjusted life year, MMR – maternal mortality ratio.

In 2021, national ASIR of maternal disorders varied widely, from 724.3 to 13 719.0 cases per 100 000 population. The highest ASIR were in South Sudan (ASIR = 13 719.0), Niger (ASIR = 13 450.9), and Chad (ASIR = 11 969.8), while the lowest were in the Republic of Korea (ASIR = 724.3), Singapore (ASIR = 946.7), and Taiwan (Province of China) (ASIR = 1383.7). National MMR ranged from 1.4 to 564.1 per 100 000 live births, with Liberia (MMR = 564.1), Central African Republic (MMR = 503.5), and Djibouti (MMR = 501.5) reporting the highest rates, and Slovenia (MMR = 1.4), Poland (MMR = 1.8), and Norway (MMR = 1.8) the lowest (**Figure 1**, Panels A and C; Tables S2 and S3 in the **Online Supplementary Document**). There was also variation in ASDR, with Chad (ASDR = 4608.9), Somalia (ASDR = 4239.5), and South Sudan (ASDR = 3684.7) at the high end, and Singapore (ASDR = 10.5), Andorra (ASDR = 12.1), and Italy (ASDR = 12.5) at the low end (**Figure 1**, Panel E; Table S4 in the **Online Supplementary Document**).

Between 1990 and 2021, significant changes in ASIR were observed across countries: Saudi Arabia (−73.5%), Libya (−73.0%), and Syria (−71.8%) saw the largest decreases, while Samoa (7.4%), Niger (4.4%), and Switzerland (4.3%) experienced increases. For MMR, Estonia (−90.8%), Slovenia (−89.5%), and Poland (−88.8%) showed the largest declines, whereas Dominica (164.2%), American Samoa (145.8%), and Canada (130.8%) had the largest increases (**Figure 1**, Panels B and D; Tables S2 and S3 in the **Online Supplementary Document**). In terms of DALYs, the Maldives (−92.9%), Jordan (−89.0%), and Syria (−88.9%) saw the largest decreases, while increases were highest in the USA (30.0%), Canada (20.8%), and Niue (16.8%) (**Figure 1**, Panel F; Table S4 in the **Online Supplementary Document**).

### Maternal disorders in different age groups

From 1990 to 2021, the incidence, mortality, and DALY rates decreased globally, but significant variations were observed among the different age groups. The global incidence of maternal disorders peaked slightly in the 15–19 age group, gradually declined, and began to rise rapidly after age 30–34. Similarly, maternal mortality and DALY rates were higher in the 10–14 age group, decreased with age, but started to rise rapidly again after age 30–34 (Figure S2, Panels A–C in the **Online Supplementary Document**). However, the mortality and DALY rates due to ectopic pregnancy remained higher after the 40–44 age group in 2021 than that in 1990. Additionally, mortality and DALY rates due to maternal deaths aggravated by HIV/AIDS were higher in the 30–34 age group in 2021 than in 1990.

### Time trends by global and SDI regions, 1990–2021

From 1990 to 2021, there was a significant decrease in the incidence, mortality, and DALY rates of maternal disorders worldwide (Figure S3 in the **Online Supplementary Document**). Although the rates were higher in the low-SDI regions than those in the high-SDI regions, they declined more rapidly in the low-SDI regions. However, maternal deaths aggravated by HIV/AIDS showed a slow upward trend in regions outside the low-SDI, with high-SDI regions showing a significant increase in mortality due to late maternal deaths.

### Association with the SDI

**Figure 2:**
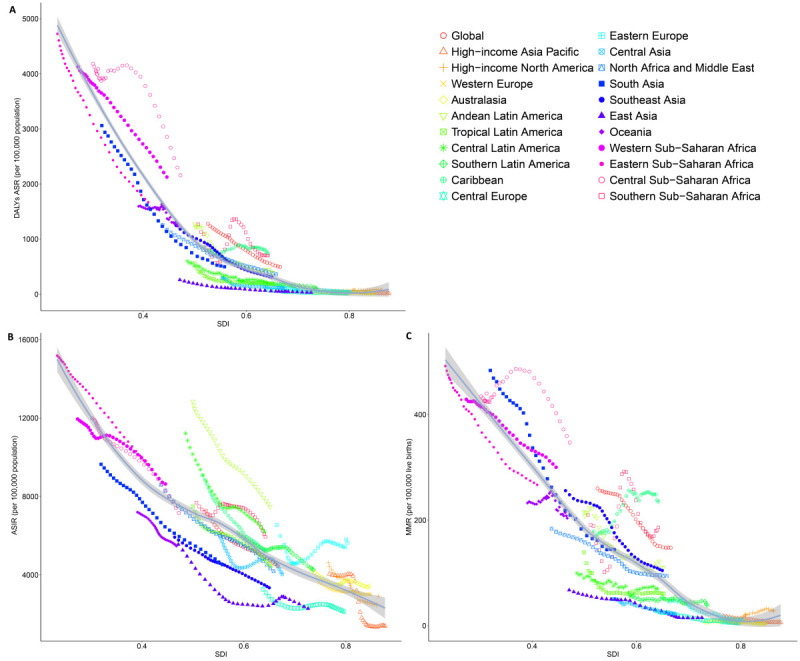
Maternal disorder incidence, mortality, and DALY rates for the 21 global burdens of disease regions by SDI, 1990–2021. **Panel A.** DALYs ASR. **Panel B.** ASIR. **Panel C.** MMR. 32 points were plotted for each region, showing the observed incidence, mortality, and DALY rates for the period. The solid line represents an adaptive association fitted with adaptive Loess regression based on all data points. The shaded area indicates the 95% CI of the expected values. Regions above the solid line showed a higher-than-expected burden, and regions below the line showed a lower-than-expected burden. ASIR – age-standardised incidence rate, ASR – age-standardised rate, CI – confidence interval, DALY – disability-adjusted life year, MMR – maternal mortality ratio, SDI – sociodemographic index.

At the regional level, an L-shaped association was identified between the SDI and the ASDR of maternal disorders from 1990 to 2021 (**Figure 2**, Panel A). The ASDRs declined markedly with increasing SDI. The ASDRs in Central sub-Saharan Africa were higher than expected based on the region's SDI from 1990 to 2021. Conversely, South Asia, Central Asia, East Asia, Eastern sub-Saharan Africa, Central Latin America, and tropical Latin America exhibited lower-than-expected burdens.

Regarding ASIR, Andean Latin America, Central Latin America, Eastern Europe, and Southern sub-Saharan Africa exhibited higher-than-expected incidence rates, while South Asia, Southeast Asia, East Asia, Oceania, and Central Europe showed lower-than-expected rates. The relationship between the MMR and SDI reflected that of DALY rates (**Figure 2**, Panels B and C).

### APC model on maternal disorders incidence, mortality, and DALYs

**Figure 3:**
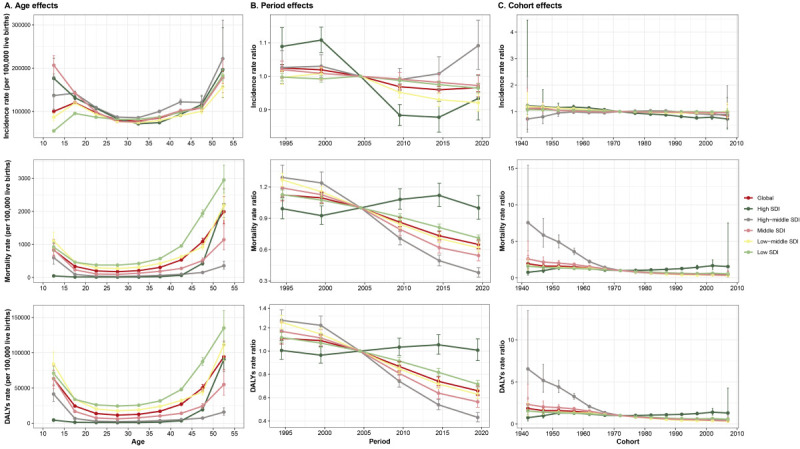
Age, period, and cohort effects on maternal disorder incidence, mortality, and DALY rates by SDI quintiles. **Panel A.** Age effects. **Panel B.** Period effects. **Panel C.** Cohort effects. Shaded areas represent the 95% CIs. CI – confidence interval, DALY – disability-adjusted life year, SDI – sociodemographic index.

Generally, similar patterns of initially decreasing and increasing age effects on mortality and DALY rates were observed across all SDI regions. Low-SDI regions exhibited higher overall mortality and DALY rates across all age groups than the other SDI regions. In high and middle-SDI regions, incidence risk decreased with age before increasing again, while other SDI regions showed a small peak in incidence risk at ages 15–19 (**Figure 3**, Panel A). Period effects showed a global decrease in maternal disorder incidence over time. However, an upward trend was observed in the high- and high-middle-SDI regions after 2012. Maternal mortality and disability declined globally, with a slight upward trend observed in high-SDI regions after 2002, followed by a stable period. This indicates the effective long-term control of risks due to maternal disorders. Globally, cohort effects showed a slow decline in incidence, mortality, and DALY rates in successive birth cohorts (**Figure 3**, Panels B and C), with a more pronounced decline in high-middle-SDI regions in the pre-1972 birth cohort.

### Trends of incidence and mortality predicted by BAPC

The BAPC model indicated that the ASIR was predicted to decline steadily from 4471.2 per 100 000 population in 2021 to 3782.2 (95% confidence interval (CI) = 3030.9–4533.5) by 2030 and further to 3403.2 (95% CI = 2123.8–4682.6) by 2036. The MMR is expected to decrease slowly from 147.8 per 100 000 live births in 2021 to 136.5 (95% CI = 100.2–172.7) by 2030 and further to 128.8 (95% CI = 63.8–193.7) by 2036 (Figure S4, Panels A and B and Tables S5 and S6 in the **Online Supplementary Document**). These projections suggest a continued decrease in the incidence and mortality of maternal disorders from 2022 to 2036, reflecting ongoing improvements in maternal health globally.

## DISCUSSION

We analysed the global burden and trends of maternal disorders from 1990 to 2021 using the GBD 2021 database. Despite significant global progress, substantial regional disparities remain. Haemorrhage and hypertensive disorders are the leading causes of maternal mortality. Notably, maternal mortality and DALYs associated with HIV/AIDS have increased, particularly among women aged 30–34 years. Projections indicate that without accelerated efforts, current progress will not meet the SDG targets for maternal mortality by 2030.

Globally, from 1990 to 2021, maternal disorder incidence, mortality, and DALYs generally declined, with pronounced decreases in regions, such as high-income Asia Pacific, East Asia, South Asia, Central Europe, and Eastern Europe. Conversely, high-income North America showed an upward trend. Previous research has largely overlooked this issue [16,38], although some studies point to contributing factors in high-income settings such as delayed childbearing, increasing maternal age, and the rising prevalence of chronic conditions such as obesity, diabetes and hypertension [39,40]. Although these issues exist globally, their higher prevalence in developed areas may be related to the observed increase in North America.

The global burden of maternal disorders has generally trended downward; however, regions in sub-Saharan Africa continue to bear the heaviest burden, which remains consistent with previous research findings [21]. Efforts in this region include training community health workers, establishing maternal health programmes, and building health facilities. Nevertheless, challenges persist, including a shortage of obstetricians, healthcare facilities, and the low socioeconomic status of women in the region [20,41–44]. These findings highlight the continued need to strengthen maternal health systems in high-burden regions.

We found that maternal haemorrhage and hypertensive disorders were associated with the highest mortality and DALY rates, consistent with previous World Health Organization reviews [45]. Despite effective interventions over recent decades, haemorrhage remains a primary contributor to maternal disorders [13,14,17]. Most obstetric haemorrhage-related deaths occur within 24 hours postpartum, often due to uterine atony, requiring immediate skilled care [46], availability of safe blood transfusions, trained personnel, and essential medications [47].

Maternal disorder burdens showed age-specific patterns, with relatively higher burdens observed in younger and older age groups. However, Huang and colleagues [38] found that the use of population-based denominators produced results that differed from this pattern. We consider that using per 100 000 population as the denominator may obscure the true burden of maternal disorders during the peak reproductive years, whereas expressing selected indicators per live birth may improve interpretability across age groups. Nevertheless, this approach should be regarded as a standardised comparative measure rather than an exact estimate of risk per pregnancy. Mortality and DALY rates aggravated by HIV/AIDS significantly increased, particularly among the 30–34 age group, due to high HIV/AIDS prevalence in certain regions and insufficient antiretroviral therapy (ART) coverage. Sociocultural factors and limited healthcare infrastructure also hinder ART implementation in resource-poor countries, such as South Africa, where ART coverage is relatively high, but uptake among pregnant women remains low [48,49]. Strengthening HIV/AIDS prevention and control and reducing discrimination are essential to mitigate AIDS-related maternal mortality.

Under the specified APC model constraints, the estimated age pattern suggested that the incidence, mortality, and DALY rates of maternal disorders first decreased and then increased with age. In low-SDI regions, adolescent mothers may be more vulnerable due to limited access to prenatal care, family planning, and health education, increasing the risk of pregnancy complications [20,50–52]. Addressing this vulnerability requires improving access to reproductive health services and education for young women in these areas. However, estimates for the youngest age group (10–14 years) should be interpreted with caution, as maternal events in this age category are rare and GBD estimates may be unstable due to small denominators and modelling uncertainty. After the age of 35–39 years, the incidence, mortality, and DALY rates gradually increased in all SDI regions, reflecting the growing risks associated with advanced maternal age, such as gestational diabetes and hypertension [53]. The trend towards delayed childbearing in certain regions driven by social and economic factors further magnifies these risks [54]. This highlights the need for comprehensive prenatal care to reduce the risk of diseases associated with advanced maternal age while strengthening health education on the risks of delayed childbearing. The period effects showed a global decline in maternal disorder risks over time; however, since 2012, high- and high-middle SDI regions have experienced a reversal in this trend, likely related to the increasing burden of chronic diseases and ageing populations. Conversely, low-SDI regions showed limited progress, highlighting the importance of expanding basic healthcare services to address maternal health disparities [18]. Cohort effects demonstrated a gradual reduction in risks across birth cohorts, particularly in high-middle-SDI regions pre-1972, underscoring generational improvements in healthcare and socioeconomic conditions.

The BAPC model projections suggest a steady decline in global maternal disorders over the next 15 years, indicating continued improvements in maternal health. However, at the current level of maternal healthcare infrastructure and the observed trend of MMR, it is projected that MMR will be 136.5 (95% CI = 100.2–172.7) per 100 000 live births by 2030, which falls short of the SDG target of 70 deaths per 100 000 live births globally [4]. According to the UN inter-agency estimates, achieving this target would require a compound annual reduction rate of >10% [11]. However, current projections suggest that the compound annual reduction rate will remain <1%. This emphasises the potential gap between the projected improvements and the ambitious targets set by the SDGs. Although the UN and GBD estimates are derived from different modelling frameworks, this benchmark provides a useful reference for illustrating the magnitude of reduction required to meet the SDG target. Uncertainties in healthcare systems and socioeconomic conditions can further influence the trajectory of these improvements. Several factors, including economic downturns, armed conflict, healthcare crises, and the emergence of new diseases, may hinder the advancement of maternal health. Moreover, the rate of decline varied across different SDI regions, with low-SDI regions exhibiting a slower rate of improvement due to constrained healthcare resources. Therefore, if the 2030 SDG targets are to be met, global efforts to improve maternal health must be strengthened urgently and substantially.

Addressing the global burden of maternal mortality is challenging but not impossible. Many countries are currently stalled in their maternal mortality transition due to slowed social and health system development [55]. The global burden of maternal mortality is concentrated in a few countries, many of which are experiencing significant shifts in environmental, economic, political, and technological contexts [55]. Greater international and national investment is needed to support maternal health improvements in these settings, particularly by addressing broader social determinants of health, including economic marginalisation and inequalities related to gender, race, and social status [56,57]. Strengthening health systems through universal health coverage and ensuring access to quality-assured medical commodities remain key strategies for reducing maternal mortality in high-burden countries [58].

The strength of this study lies in our up-to-date and comprehensive analysis of the global burden of maternal disorders from 1990 to 2021. Beyond describing global and regional patterns, we integrated an APC analysis with forward projections to characterise temporal dynamics and future trajectories, while also assessing the gap between projected trends and the SDG targets for maternal mortality. Given the specific epidemiological characteristics of maternal disorders, age-specific incidence and DALY rates based on female population denominators may be influenced by variations in fertility patterns across age groups. To improve interpretability across reproductive ages, we additionally expressed selected age-specific incidence and DALY indicators per 100 000 live births using ASFR data from GBD 2021. This transformation was intended to provide a standardised comparative measure of maternal burden across age groups rather than to replace official GBD metrics.

This study has several limitations. We relied on estimates from the GBD 2021 database rather than directly observed epidemiological data, and data availability and quality vary across regions, particularly in low-SDI settings, which may affect the stability of some estimates. In addition, GBD estimates are derived from complex modelling procedures, and using these estimates as inputs for subsequent APC and BAPC analyses may reinforce underlying modelling assumptions, while full uncertainty propagation cannot be achieved when only publicly available summary estimates are used. Moreover, we rescaled ASIRs and ASDRs using ASFR and expressed them per 100 000 live births to improve interpretability across reproductive ages. However, live births do not fully capture all pregnancy exposures, particularly for outcomes such as abortion, miscarriage, and ectopic pregnancy. These transformed indicators should therefore be interpreted as standardised comparative measures rather than exact estimates of risk per pregnancy. Finally, we estimated the projected live births using the ASFR observed in 2021. This approach provides a conditional demographic scenario for forecasting but does not account for future changes in fertility patterns. As a result, the projected mortality trends should be interpreted with caution, particularly in settings undergoing rapid fertility transition.

## CONCLUSIONS

While the global burden of maternal disorders is decreasing, notable regional disparities remain. Higher-SDI regions face a lower burden, whereas sub-Saharan Africa experiences a considerably higher burden. Maternal haemorrhage and hypertensive disorders continue to be the primary causes of maternal mortality, with increasing deaths from HIV/AIDS, particularly among women aged 30–34 and older. Considering the current state of maternal healthcare and mortality trends, achieving the SDG targets by 2030 seems implausible, given a substantial shortfall in the necessary annual reductions in maternal mortality. It is crucial to reinforce maternal care strategies, collaborate to eliminate preventable maternal mortality, as well as intensify efforts to improve the coverage and equity of quality maternal health services to meet the SDG targets.

## Additional material


Online Supplementary Document
