# Supplementary material for: Global burden of maternal disorders, 1990–2021: insights from the Global Burden of Disease Study 2021 and challenges for achieving 2030 Sustainable Development Goals
Source: J Glob Health. 2026 May 15;16:04147. doi: 10.7189/jogh.16.04147 (PMC13178056; doi:10.7189/jogh.16.04147)
Supplement: Online Supplementary Document [file jogh-16-04147-s001.pdf]

Supplement to: Zhu J, Ni C, Wei S, Bi J, Wang X, Zheng J, Xu X, Lv W, Cai W, Dong Z, Wu Y, Liu H, Wang B. Global burden of maternal disorders, 1990–2021: insights from the Global Burden of Disease Study 2021 and challenges for achieving 2030 Sustainable Development Goals. J Glob Health. 2026;16:04147.

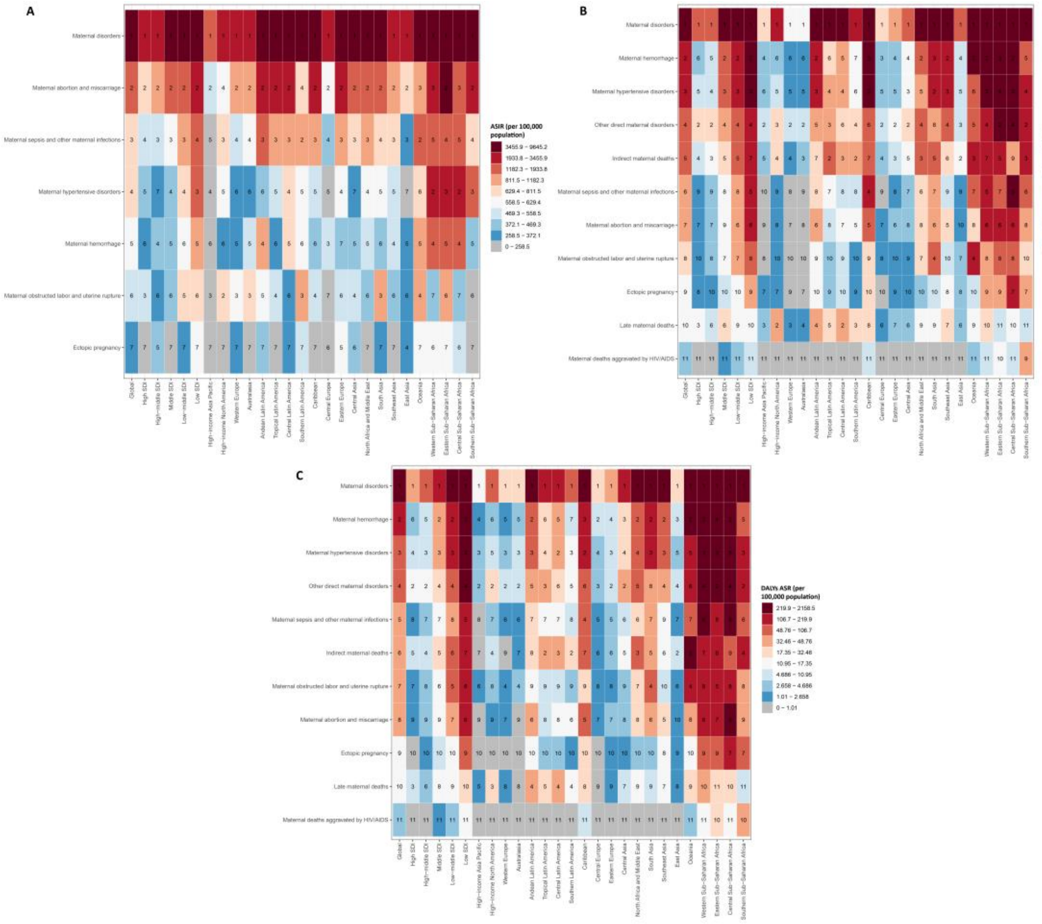

Figure S1. Global age-standardised rates of incidence, mortality and DALYs for maternal disorders by region and SDI in 2021. **Panel A.** ASIR. **Panel B.** MMR. **Panel C.** DALYs ASR. ASIR – age standardized incidence rate, MMR – maternal mortality ratio, ASR – age-standardised rate, DALYs – disability-adjusted life years, SDI – sociodemographic index.

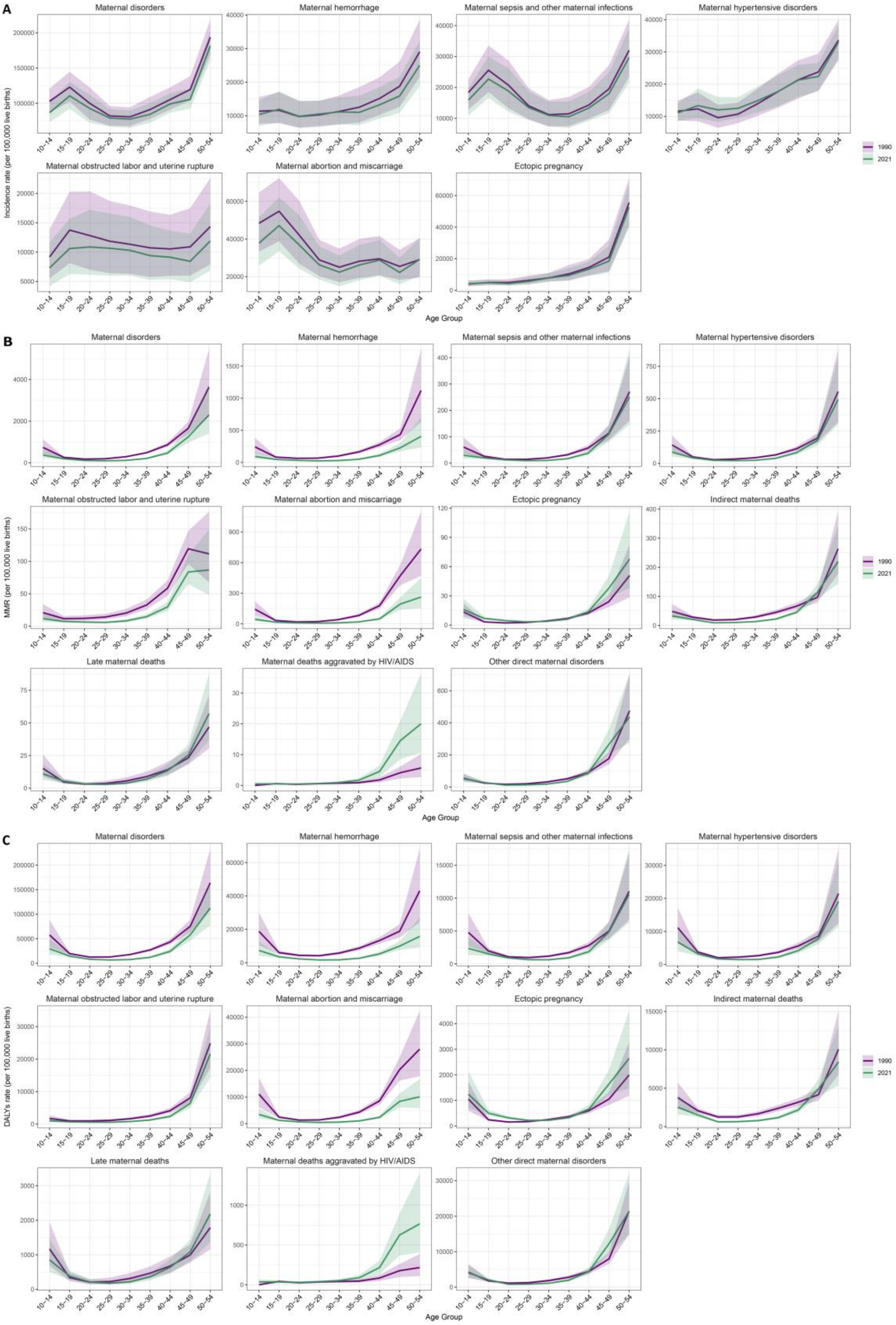

Figure S2. Maternal disorder incidence, mortality, and DALY rates by age group for 1990 and 2021. **Panel A.** Incidence. **Panel B.** MMR. **Panel C.** DALYs. MMR – maternal mortality ratio, DALYs – disability-adjusted life years.

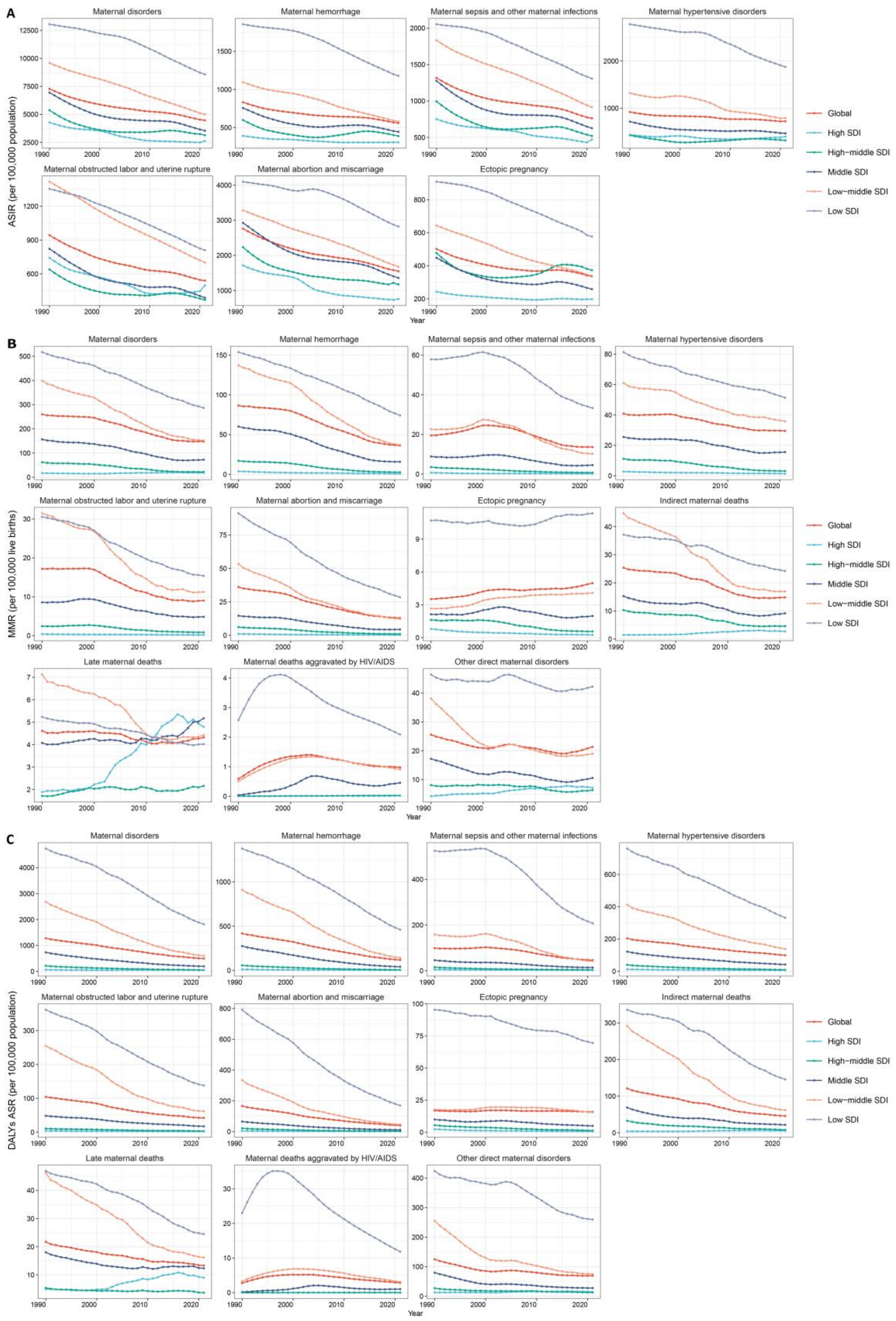

Figure S3. Maternal disorders incidence, mortality, and DALYs rates by global and SDI regions, from 1990 to 2021. **Panel A.** ASIR. **Panel B.** MMR. **Panel C.** DALYs ASR. ASIR – age-standardised incidence rate, MMR – maternal mortality ratio, DALYs – disability-adjusted life years, ASR – age-standardised rate.

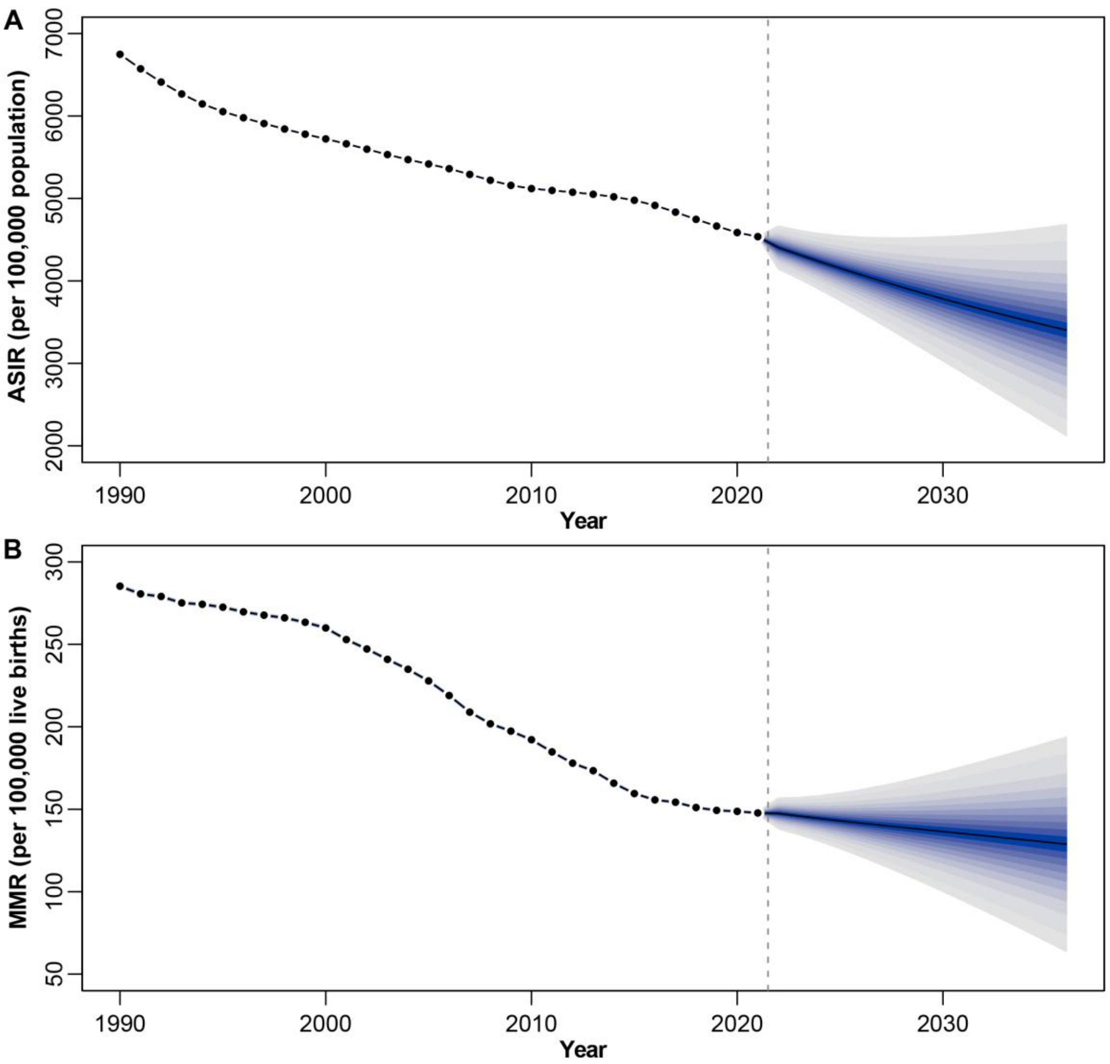

Figure S4. Trends of incidence and mortality for maternal disorders globally from 2022 to 2036 predicted by BAPC model. **Panel A.** ASIR. **Panel B.** MMR. Trends in observed (black dots) and predicted (solid lines) ASR are shown, with shading that intensifies from the inside to the outside representing the 5–95% confidence intervals. ASR – age-standardised rate, ASIR – age-standardised incidence rate, MMR – maternal mortality ratio, BAPC – Bayesian age-period-cohort.

Table S1. Adherence to JoGH's GRABDROP guidelines items.

**JoGH guideline items**
1. Please list all papers published by each co-author in previous 3 years that were based on secondary analysis of a big data repository
[1] Lv W, Wang S, Duan L, et al. Estimating the global macroeconomic impact of colorectal cancer: evidence from Global Burden of Disease 2021 and Value of a Statistical Life Year framework. *Int J Surg*. Published online February 5, 2026.<br>[2] Guan SY, Zheng JX, Sam NB, Xu S, Shuai Z, Pan F. Global burden and risk factors of musculoskeletal disorders among adolescents and young adults in 204 countries and territories, 1990-2019. *Autoimmun Rev*. 2023;22(8):103361.<br>[3] Zhang SX, Wang JC, Yang J, et al. Epidemiological features and temporal trends of the co-infection between HIV and tuberculosis, 1990-2021: findings from the Global Burden of Disease Study 2021. *Infect Dis Poverty*. 2024;13(1):59. Published 2024 Aug 16.<br>[4] Zheng JX, Liu Y, Guan SY, et al. Global, regional, and national burden of neglected tropical diseases and malaria in the general population, 1990-2021: Systematic analysis of the global burden of disease study 2021. *J Adv Res*. 2026;79:769-781.<br>[5] Guan SY, Zheng JX, Feng XY, et al. The impact of population ageing on musculoskeletal disorders in 204 countries and territories, 1990-2021: global burden and healthcare costs. *Ann Rheum Dis*. 2025;84(12):2128-2138.<br>[6] Wei S, Zhu J, Ni C, et al. Tackling global inequalities in maternal hypertensive disorders: trends and the impact of public health emergencies, 1990-2021. *Front Public Health*. 2025;13:1696754.
2. Please explain the key elements of your study design and the use of the available datasets that make your study an original scientific contribution
This study is a secondary ecological analysis based on publicly available estimates from the Global Burden of Disease (GBD) 2021 database. Its original contribution includes the following elements:<br>**1)** This study used the most recent GBD 2021 estimates to provide an updated assessment of maternal disorders at the global and regional levels from 1990 to 2021.<br>**2)** Given the specific epidemiological characteristics of maternal disorders, age-specific incidence and DALY rates based on female population denominators may be influenced by variations in fertility patterns across age groups. To improve interpretability across reproductive ages, this study additionally expressed selected age-specific incidence and DALY indicators per 100,000 live births using age-specific fertility rate (ASFR) data from GBD 2021.<br>**3)** This study applied age–period–cohort (APC) analysis to the GBD estimates to examine the distribution of maternal disorder burden across age, period, and cohort dimensions. This extended the use of the dataset from descriptive trend reporting to temporal decomposition analysis.<br>**4)** This study further used Bayesian APC projections to estimate future trends in incidence and mortality, and to assess the gap between the projected maternal mortality ratio (MMR) and the Sustainable Development Goal target. Because MMR is expressed per live births, its projection required combining projected population counts with age-specific fertility patterns. For this reason, projected live births were estimated using the ASFR observed in 2021, allowing MMR to be forecast under a conditional fertility scenario.<br>Together, these elements extend the use of publicly available GBD data beyond descriptive reporting and provide an updated, methodologically enriched, and policy-relevant assessment of maternal disorders.

3. Please list all publications that addressed similar research questions in the same dataset and indicate where you cited them in your paper

The main publications addressing similar questions using the GBD dataset and cited in our manuscript are:

[1] GBD 2019 Pakistan Collaborators. The state of health in Pakistan and its provinces and territories, 1990-2019: a systematic analysis for the Global Burden of Disease Study 2019. The Lancet Global health. 2023;11(2): e229-43. (**Ref. 12, cited in Introduction**)

[2] Montoya A, Lozano R, Sanchez-Dominguez M, Fritz J, Lamadrid-Figueroa H. Burden, incidence, mortality and lethality of maternal disorders in mexico 1990-2019: an analysis for the Global Burden of Disease Study 2019. Archives of Medical Research. 2023;54(2):152-9. (**Ref. 13, cited in Introduction and Discussion**)

[3] Sepanlou SG, Rezaei Aliabadi H, Malekzadeh R, Naghavi M. Levels and trends of maternal mortality and morbidity by cause in North Africa and Middle East, 1990 to 2019: an analysis for the Global Burden of Disease Study 2019. Archives of Iranian medicine. 2022;25(10):666-75. **(Ref. 14, cited in Introduction and Discussion)**

[4] GBD 2015 Maternal Mortality Collaborators. Global, regional, and national levels of maternal mortality, 1990-2015: a systematic analysis for the Global Burden of Disease Study 2015. Lancet. 2016;388(10053):1775-812. **(Ref. 16, cited in Introduction)**

[5] GBD 2015 Eastern Mediterranean Region Maternal Mortality Collaborators. Maternal mortality and morbidity burden in the Eastern Mediterranean region: findings from the Global Burden of Disease 2015 Study. International Journal of Public Health. 2018;63(Suppl 1):47-61. **(Ref. 17, cited in Introduction and Discussion)**

[6] Huang H, Ma J, Ling S, Han L, Jiang G, Xu W. Incidence and disability-adjusted life years of maternal disorders at the global, regional, and national levels from 2007 to 2017: a systematic analysis for the Global Burden of Disease Study 2017. Int J Gynecol Obstet. 2022;157(3):618-39. **(Ref. 34, cited in Discussion)**

4. Please explain how you addressed multiple testing through an appropriately rigorous statistical threshold and indicate this in the methods section

The analysis focused primarily on descriptive epidemiological patterns and model-based trend characterization rather than formal multiple hypothesis testing. Therefore, multiple-testing correction procedures were not applied. Uncertainty was presented using the 95% uncertainty intervals reported in the GBD framework.

5. Please declare to what extent have AI chatbots been used in developing your paper and to which parts of the paper did they contribute

During manuscript preparation, we used ChatGPT to refine language and improve clarity in the Introduction section. All content was critically reviewed, revised, and approved by the authors.

Table S2. Incidence of maternal disorders in 2021, and the percentage change in the ASR per 100,000 population compared to 1990, by country.

Location	Number (95% UI)	ASR per 100,000 population (95% UI)	Percentage change in the ASR (%)*
Afghanistan	816172 (696189, 965459)	8449.9 (7207.7, 9995.4)	-18.3
Albania	19492 (17060, 22367)	2499 (2187.3, 2867.7)	-39.1
Algeria	583894 (507833, 669355)	4101.1 (3566.9, 4701.4)	-37.5
American Samoa	567 (497, 639)	3565.2 (3127.9, 4019.7)	-56.2
Andorra	423 (360, 483)	1642.5 (1397.8, 1876)	-33.4
Angola	762680 (677837, 864416)	7369.9 (6550.1, 8353)	-39.7
Antigua and Barbuda	849 (737, 985)	2792.1 (2423.1, 3240.1)	-49.1
Argentina	615292 (539824, 687620)	4134.3 (3627.2, 4620.3)	-32.3
Armenia	18592 (16138, 21495)	2029.1 (1761.2, 2345.9)	-55
Australia	247634 (211680, 283239)	3239.7 (2769.3, 3705.5)	-14.3
Austria	101375 (90292, 112438)	4004.7 (3566.9, 4441.8)	-34.3
Azerbaijan	91484 (77815, 108503)	2648 (2252.4, 3140.6)	-51.7
Bahamas	4906 (4116, 5786)	3557 (2984.1, 4194.6)	-40.2
Bahrain	17463 (14722, 20873)	4311 (3634.2, 5152.7)	-55.4
Bangladesh	1897861 (1622741, 2184608)	3284.2 (2808.1, 3780.4)	-62.1
Barbados	3668 (3064, 4281)	4026.2 (3362.6, 4699.1)	-42.5
Belarus	115772 (100055, 132882)	4275.7 (3695.3, 4907.6)	-32.4
Belgium	99061 (85029, 112291)	3103.1 (2663.5, 3517.5)	-15.4
Belize	10598 (8745, 12652)	6962.5 (5745.2, 8312.2)	-57.2
Benin	491218 (433132, 559713)	11514 (10152.4, 13119.4)	1.3
Bermuda	599 (509, 718)	3429.4 (2914.1, 4110.7)	-38.9
Bhutan	10613 (8981, 12413)	4164.3 (3523.9, 4870.3)	-60
Bolivia (Plurinational State of)	321628 (271885, 371873)	8197.5 (6929.7, 9478.1)	-49.1
Bosnia and Herzegovina	16961 (14872, 19476)	1822.6 (1598.1, 2092.9)	-40.7
Botswana	38596 (34063, 43872)	4586.2 (4047.6, 5213.2)	-43.2
Brazil	3230003 (2802667, 3737119)	4420.7 (3835.8, 5114.7)	-40.6
Brunei Darussalam	2278 (1870, 2763)	1495.2 (1227.3, 1813.4)	-51.8
Bulgaria	38011 (33333, 43278)	2066.5 (1812.2, 2352.8)	-27.6
Burkina Faso	795724 (701479, 895965)	10991 (9689.3, 12375.6)	-5.3
Burundi	413738 (365204, 467764)	9942.8 (8776.5, 11241.2)	-29.5
Cabo Verde	10380 (9044, 12021)	5520.3 (4809.7, 6393.2)	-48.9
Cambodia	350473 (292480, 414622)	6125 (5111.5, 7246.1)	-24.8
Cameroon	850742 (756608, 950790)	8296.6 (7378.6, 9272.3)	-31.6
Canada	230111 (195618, 262603)	2177.8 (1851.4, 2485.3)	-10.3
Central African Republic	135331 (118530, 152206)	7458.7 (6532.7, 8388.8)	-35.1
Chad	628888 (558122, 712362)	11969.8 (10622.9, 13558.6)	-3.4
Chile	264180 (239683, 286993)	4446.7 (4034.3, 4830.7)	-29.5
China	9622007 (8472175, 10839009)	2298.5 (2023.9, 2589.2)	-59
Colombia	1095976 (919180, 1275117)	6695.7 (5615.6, 7790.2)	-17.9

Location	Number (95% UI)	ASR per 100,000 population (95% UI)	Percentage change in the ASR (%)*
Comoros	16103 (14352, 18063)	6447.7 (5746.7, 7232.6)	-53.2
Congo	93135 (82853, 104780)	5032.5 (4477, 5661.8)	-45
Cook Islands	136 (120, 156)	2426.6 (2137.2, 2779.6)	-55.5
Costa Rica	70374 (59618, 82556)	4360.3 (3693.9, 5115.1)	-53.8
Côte d'Ivoire	636287 (573096, 710740)	7383 (6649.8, 8246.9)	-31.7
Croatia	21475 (19457, 23799)	1870.5 (1694.7, 2072.9)	-27.4
Cuba	145158 (121336, 169826)	4408.8 (3685.3, 5158)	-39.2
Cyprus	11134 (9504, 13099)	2556.4 (2182, 3007.3)	-48.3
Czechia	64542 (56360, 73978)	2204.1 (1924.7, 2526.3)	-18.8
Democratic People's Republic of Korea	165685 (141256, 197462)	1949.8 (1662.3, 2323.7)	-59.6
Democratic Republic of the Congo	2057626 (1830968, 2324561)	7264 (6463.8, 8206.3)	-40.5
Denmark	55209 (46966, 63653)	3395.6 (2888.6, 3915)	-4.8
Djibouti	28648 (25655, 31701)	7133.2 (6388, 7893.3)	-37.8
Dominica	718 (626, 829)	3365.7 (2935.8, 3887.1)	-61.6
Dominican Republic	174148 (148109, 203380)	4802.5 (4084.4, 5608.6)	-53.3
Ecuador	501020 (465673, 540241)	8381.7 (7790.3, 9037.8)	-34.4
Egypt	1542926 (1325145, 1782210)	4634.7 (3980.5, 5353.5)	-40.6
El Salvador	79368 (69550, 91484)	3550.4 (3111.2, 4092.4)	-71.3
Equatorial Guinea	27971 (24919, 31215)	5965.2 (5314.4, 6657)	-50
Eritrea	216969 (187736, 246129)	10174.5 (8803.6, 11541.9)	-22.1
Estonia	20192 (17262, 23271)	5641.5 (4823, 6501.9)	-30.1
Eswatini	25802 (22346, 29664)	6482.9 (5614.6, 7453.2)	-22.4
Ethiopia	4118678 (3564743, 4683776)	11507.6 (9959.9, 13086.5)	-37.2
Fiji	14475 (12209, 16875)	4908.2 (4139.9, 5721.9)	-9.5
Finland	35208 (29767, 40626)	2417 (2043.5, 2789)	-18.8
France	610465 (518380, 698273)	3309.4 (2810.2, 3785.4)	-14.9
Gabon	32389 (28957, 36073)	5113.7 (4571.9, 5695.3)	-45.7
Gambia	59399 (53033, 66734)	7444.3 (6646.4, 8363.5)	-34.9
Georgia	33106 (28935, 37405)	3271.1 (2859, 3695.8)	-7.9
Germany	706980 (602691, 813558)	3207.6 (2734.5, 3691.2)	-5.3
Ghana	820979 (722778, 924219)	7056.8 (6212.7, 7944.2)	-33.2
Greece	65595 (57042, 74018)	2324.7 (2021.5, 2623.2)	-21.8
Greenland	583 (499, 670)	3515.9 (3007.5, 4042.5)	-33.5
Grenada	1487 (1247, 1769)	4520.2 (3790.6, 5376.9)	-56.4
Guam	2413 (2069, 2785)	5117.5 (4388.7, 5908.3)	-36.7
Guatemala	379689 (321089, 441397)	6870.8 (5810.4, 7987.4)	-51.1
Guinea	410726 (362863, 467066)	9422.5 (8324.5, 10715)	-23.5
Guinea-Bissau	50538 (45190, 56310)	7400.7 (6617.5, 8246)	-29.6
Guyana	21636 (17856, 25630)	8447.3 (6971.5, 10006.7)	-48.8
Haiti	316761 (273731, 364523)	7117.1 (6150.3, 8190.3)	-41.4
Honduras	202258 (175218, 234653)	5671.3 (4913.1, 6579.6)	-64.8

Location	Number (95% UI)	ASR per 100,000 population (95% UI)	Percentage change in the ASR (%)*
Hungary	55475 (48450, 63228)	2042.2 (1783.6, 2327.6)	-20.4
Iceland	3092 (2622, 3521)	3049.4 (2586.6, 3473.4)	-25.6
India	19415613 (16263705, 22662008)	4089.5 (3425.6, 4773.3)	-56.1
Indonesia	2581602 (2282932, 2931564)	2730.7 (2414.7, 3100.8)	-50.5
Iran (Islamic Republic of)	997756 (878635, 1138219)	3461.3 (3048.1, 3948.6)	-58.9
Iraq	618372 (531954, 711411)	4565.6 (3927.6, 5252.6)	-55.7
Ireland	37585 (31936, 43039)	2498.1 (2122.6, 2860.6)	-14.1
Israel	150905 (129504, 168986)	5272.8 (4525, 5904.5)	-9.2
Italy	389896 (354913, 427667)	2437.3 (2218.6, 2673.4)	-28.8
Jamaica	35322 (29733, 41707)	3689.4 (3105.6, 4356.3)	-58.3
Japan	554048 (493655, 629515)	1718.5 (1531.2, 1952.6)	-65.9
Jordan	152785 (131938, 176016)	3815.5 (3294.9, 4395.6)	-51.5
Kazakhstan	248603 (214196, 286636)	4101.4 (3533.8, 4728.9)	-14.5
Kenya	1251630 (1104645, 1418120)	7290.5 (6434.4, 8260.3)	-44
Kiribati	1780 (1551, 2034)	4347.6 (3789, 4968.3)	-38.2
Kuwait	37531 (32631, 43268)	2172 (1888.5, 2504.1)	-58.6
Kyrgyzstan	96581 (82562, 113385)	4353.3 (3721.4, 5110.7)	-33.4
Lao People's Democratic Republic	117085 (100571, 134549)	4705 (4041.4, 5406.8)	-44.2
Latvia	29138 (24817, 34002)	5767 (4911.7, 6729.7)	-11.9
Lebanon	50820 (43986, 58267)	2775.6 (2402.3, 3182.3)	-59.5
Lesotho	28532 (25182, 32183)	4422 (3902.8, 4987.8)	-40.6
Liberia	141890 (125275, 159661)	7880 (6957.3, 8867)	-43.6
Libya	49116 (42680, 56121)	1989.2 (1728.6, 2272.9)	-73
Lithuania	43760 (36749, 51206)	5887.8 (4944.5, 6889.7)	-23.5
Luxembourg	4471 (3807, 5223)	2288.8 (1948.8, 2673.8)	-25.6
Madagascar	669025 (589600, 754493)	7022.8 (6189.1, 7920)	-42.2
Malawi	470290 (417062, 533664)	7107.5 (6303.1, 8065.3)	-46.7
Malaysia	316644 (275957, 361545)	3031.1 (2641.6, 3460.9)	-43.3
Maldives	4553 (3946, 5202)	3226.6 (2795.9, 3686.2)	-65.6
Mali	772774 (685730, 865407)	10595.4 (9402, 11865.5)	-14.7
Malta	3076 (2613, 3540)	2630.8 (2235.1, 3028.3)	-25.5
Marshall Islands	755 (649, 864)	3977.1 (3420.6, 4551.6)	-43.4
Mauritania	83493 (76055, 92326)	5869.5 (5346.6, 6490.5)	-41.5
Mauritius	7373 (6429, 8441)	1854.2 (1617, 2122.9)	-50.5
Mexico	2861714 (2525913, 3211363)	6448 (5691.3, 7235.8)	-48.4
Micronesia (Federated States of)	1218 (1064, 1382)	3590.7 (3137.2, 4074.4)	-45.4
Monaco	234 (202, 265)	2445.8 (2111.5, 2773)	-16.7
Mongolia	50797 (44177, 58477)	4694.8 (4082.9, 5404.5)	-35
Montenegro	4530 (3935, 5101)	2480.4 (2154.5, 2793.2)	-24.2
Morocco	362951 (315647, 413779)	2963.1 (2576.9, 3378.1)	-57.8
Mozambique	1004873 (892244, 1144767)	9934.4 (8820.9, 11317.4)	-28.6

Location	Number (95% UI)	ASR per 100,000 population (95% UI)	Percentage change in the ASR (%)*
Myanmar	574742 (502383, 659739)	2980.6 (2605.3, 3421.4)	-43
Namibia	50719 (43918, 58518)	6005.8 (5200.5, 6929.4)	-38.9
Nauru	200 (175, 230)	5502.3 (4807.7, 6323.1)	-33.1
Nepal	826878 (678293, 969458)	7336.3 (6018, 8601.3)	-34.4
Netherlands	138487 (116745, 159860)	2908.2 (2451.6, 3357)	-19
New Zealand	65939 (60077, 72019)	4319.6 (3935.5, 4717.9)	-36.9
Nicaragua	98463 (86806, 111871)	4314.1 (3803.4, 4901.6)	-65
Niger	984272 (871016, 1100327)	13450.9 (11903.2, 15036.9)	4.4
Nigeria	6265817 (5665678, 6939794)	8152.4 (7371.5, 9029.3)	-34.3
Niue	15 (13, 17)	3026.8 (2643.3, 3447)	-34.8
North Macedonia	14075 (12163, 15915)	2127.2 (1838.3, 2405.3)	-44.9
Northern Mariana Islands	405 (357, 459)	2730.6 (2406.8, 3097.1)	-48.5
Norway	47717 (39177, 57310)	3070.4 (2520.8, 3687.7)	-20.9
Oman	44048 (38481, 50740)	3539.7 (3092.4, 4077.5)	-54.9
Pakistan	5943792 (5149259, 6830076)	7686.1 (6658.6, 8832.1)	-40.6
Palau	120 (105, 137)	2363.9 (2073.4, 2695.9)	-40.2
Palestine	69810 (59165, 80911)	4131.9 (3501.9, 4789)	-56.8
Panama	89110 (74157, 106744)	6504.7 (5413.2, 7792)	-36.8
Papua New Guinea	184975 (160957, 211444)	5563.3 (4840.9, 6359.4)	-24.9
Paraguay	121808 (103499, 142033)	5105.6 (4338.1, 5953.3)	-44.8
Peru	811624 (695553, 941150)	6774.5 (5805.7, 7855.6)	-43.3
Philippines	2152604 (1893733, 2464894)	5750.8 (5059.2, 6585.1)	-33.7
Poland	208954 (187360, 233445)	1900.3 (1703.9, 2123)	-49.8
Portugal	67170 (61227, 74108)	2253.5 (2054.2, 2486.3)	-17.9
Puerto Rico	21844 (18343, 25804)	2282.4 (1916.6, 2696.2)	-64.8
Qatar	19736 (17205, 22334)	3006.3 (2620.9, 3402.2)	-57.3
Republic of Korea	108268 (87803, 131060)	724.3 (587.4, 876.8)	-55.7
Republic of Moldova	43948 (37750, 50270)	4025.3 (3457.6, 4604.3)	-47.1
Romania	140094 (123932, 156925)	2607.5 (2306.7, 2920.8)	-21
Russian Federation	2655373 (2300545, 3030640)	6185.6 (5359, 7059.7)	-5.7
Rwanda	296196 (264857, 333030)	6552 (5858.8, 7366.8)	-48.8
Saint Kitts and Nevis	714 (594, 851)	3674.9 (3058.5, 4378.5)	-61.6
Saint Lucia	1842 (1575, 2173)	3195.9 (2732.1, 3770.8)	-67.5
Saint Vincent and the Grenadines	2034 (1672, 2404)	5706.7 (4691.1, 6747.4)	-49.2
Samoa	3271 (2877, 3752)	5080.6 (4468.1, 5827.1)	7.4
San Marino	176 (148, 201)	1864.8 (1571.3, 2130)	-23.7
Sao tome and Principe	3196 (2843, 3604)	4369.7 (3887, 4927.7)	-54.1
Saudi Arabia	286290 (250769, 327417)	2376.2 (2081.4, 2717.6)	-73.5
Senegal	297578 (266615, 332339)	5839.4 (5231.8, 6521.5)	-45
Serbia	50154 (43446, 57467)	1960.3 (1698.1, 2246.2)	-31
Seychelles	1157 (1003, 1326)	3677.6 (3187.3, 4215.6)	-31.7

Location	Number (95% UI)	ASR per 100,000 population (95% UI)	Percentage change in the ASR (%)*
Sierra Leone	226775 (202514, 256472)	7772 (6940.5, 8789.7)	-35.9
Singapore	16695 (13507, 20423)	946.7 (765.9, 1158.2)	-53.5
Slovakia	36557 (31671, 41629)	2318.6 (2008.7, 2640.3)	-34
Slovenia	13202 (11449, 15122)	2421.5 (2100, 2773.6)	-9.3
Solomon Islands	12838 (11233, 14606)	5800 (5074.9, 6598.7)	-35.5
Somalia	736524 (656313, 825788)	11544.5 (10287.2, 12943.7)	-19.6
South Africa	1108251 (939306, 1303548)	5717.2 (4845.7, 6724.7)	-19.5
South Sudan	428029 (376786, 485945)	13719 (12076.6, 15575.3)	-4.2
Spain	324373 (288262, 361804)	2514.7 (2234.7, 2804.9)	-18.4
Sri Lanka	176674 (153801, 203187)	2439.9 (2124, 2806.1)	-33
Sudan	838453 (713080, 977412)	5773.4 (4910.1, 6730.2)	-50.5
Suriname	8425 (7133, 9836)	4479.4 (3792.3, 5229.4)	-42.3
Sweden	86785 (74953, 100614)	3071.5 (2652.7, 3560.9)	-35
Switzerland	79866 (68705, 91059)	3201.7 (2754.3, 3650.4)	4.3
Syrian Arab Republic	114740 (100312, 130197)	2246.3 (1963.8, 2548.9)	-71.8
Taiwan (Province of China)	96848 (83781, 111424)	1383.7 (1197, 1591.9)	-51.4
Tajikistan	158580 (133208, 188262)	4880.5 (4099.7, 5794)	-41.4
Thailand	442521 (375375, 523671)	2107.4 (1787.7, 2493.9)	-44.2
Timor-Leste	24381 (21192, 28215)	5445.2 (4732.9, 6301.5)	-44.2
Togo	193694 (172255, 217557)	6946.8 (6177.9, 7802.6)	-30.6
Tokelau	15 (13, 17)	3609 (3160.8, 4090.2)	-33.9
Tonga	1655 (1441, 1875)	4988.4 (4343.1, 5651.9)	-38
Trinidad and tobago	16820 (14362, 19635)	3929.2 (3355, 4586.8)	-45.7
Tunisia	115982 (98612, 138968)	3005.2 (2555.1, 3600.7)	-64.9
Türkiye	996955 (841779, 1152944)	3681.1 (3108.1, 4257)	-58.4
Turkmenistan	69784 (59522, 83248)	4332.1 (3695, 5167.9)	-38.8
Tuvalu	178 (153, 203)	4760.1 (4087.5, 5412.8)	-43
Uganda	1366490 (1193753, 1543315)	9882.5 (8633.3, 11161.3)	-40.7
Ukraine	539535 (467005, 617533)	4188.9 (3625.7, 4794.4)	-33.3
United Arab Emirates	47972 (41610, 55120)	2321.2 (2013.3, 2667)	-63.1
United Kingdom	607087 (534725, 685525)	3077.3 (2710.5, 3474.9)	-22.3
United Republic of Tanzania	1841818 (1605021, 2115897)	9467.9 (8250.6, 10876.8)	-33.7
United States of America	2898279 (2634615, 3183021)	2998.5 (2725.7, 3293.1)	-38.1
United States Virgin Islands	1122 (934, 1320)	4886.8 (4070, 5750.4)	-51.6
Uruguay	51780 (46007, 58335)	4940.9 (4390.1, 5566.5)	-13.3
Uzbekistan	473045 (400082, 560127)	4241.5 (3587.3, 5022.4)	-43.4
Vanuatu	6401 (5552, 7482)	6344.7 (5503, 7416)	-36.5
Venezuela (Bolivarian Republic of)	446986 (384127, 518725)	5094 (4377.6, 5911.5)	-39.1
Viet Nam	988028 (874454, 1108729)	3054 (2702.9, 3427.1)	-57.5
Yemen	666412 (576850, 772636)	6084.6 (5266.9, 7054.5)	-51
Zambia	634210 (547328, 726180)	9860.7 (8509.8, 11290.6)	-39.3

Location	Number (95% UI)	ASR per 100,000 population (95% UI)	Percentage change in the ASR (%)*
Zimbabwe	374094 (322047, 427932)	7087.2 (6101.1, 8107.1)	-25.7

ASR — age-standardised rate, UI — uncertainty intervals.

*Percentage change for each metric is in comparison to the corresponding point estimate in 1990.

Table S3. Deaths due to maternal disorders in 2021, and the percentage change in the MMR per 100,000 live births compared to 1990, by country.

Location	Number (95% UI)	MMR per 100,000 live births (95% UI)	Percentage change in the MMR (%)*
Afghanistan	3689 (2144, 5677)	304.8 (177.8, 470.5)	-41.7
Albania	2 (1, 3)	6.5 (3.9, 10.5)	-76.7
Algeria	584 (352, 881)	64.4 (38.9, 97.5)	-60.2
American Samoa	1 (1, 2)	123 (71, 198.6)	145.8
Andorra	0 (0, 0)	3.7 (1.8, 6.7)	-45
Angola	2216 (1282, 3562)	184.2 (108, 288.6)	-60.4
Antigua and Barbuda	0 (0, 1)	41.3 (34.9, 49.1)	37.1
Argentina	243 (182, 318)	45.2 (34.4, 58.8)	-31.2
Armenia	4 (3, 5)	11.4 (8.8, 14.5)	-65.1
Australia	9 (7, 11)	3 (2.2, 3.8)	-52.7
Austria	2 (2, 3)	2.6 (2.2, 3.1)	-60.3
Azerbaijan	22 (15, 32)	16.2 (10.6, 23.3)	-56.5
Bahamas	4 (3, 5)	104.1 (76.7, 140.1)	89.4
Bahrain	6 (4, 9)	35.2 (22.3, 52.2)	-24.6
Bangladesh	4165 (2811, 5817)	148.5 (101.8, 204.8)	-70.6
Barbados	2 (1, 2)	59.5 (44.2, 78.8)	26.3
Belarus	7 (5, 11)	9.1 (6.5, 12.6)	-66.4
Belgium	4 (3, 4)	3.1 (2.5, 3.7)	-53.4
Belize	5 (5, 7)	72.6 (60, 88.7)	129.4
Benin	1192 (726, 1718)	228.2 (138.5, 336.9)	-54.1
Bermuda	0 (0, 0)	37 (29, 46.3)	9.5
Bhutan	20 (10, 33)	154.4 (82.1, 266.7)	-71.5
Bolivia (Plurinational State of)	494 (352, 706)	201.6 (141.2, 283.7)	-43
Bosnia and Herzegovina	1 (1, 2)	4.4 (2.7, 6.5)	-78.8
Botswana	38 (23, 54)	78.3 (46.1, 113.7)	-43.4
Brazil	1987 (1806, 2192)	59.6 (54.2, 65.9)	-57.4
Brunei Darussalam	1 (1, 2)	21.5 (13.3, 34.2)	-51
Bulgaria	4 (3, 6)	7.4 (5.6, 9.4)	-75.3
Burkina Faso	2023 (1371, 2915)	212.7 (143.5, 304.8)	-31.7
Burundi	1337 (823, 1925)	285.2 (176.9, 414.3)	-56.5
Cabo Verde	5 (3, 9)	62 (34.5, 102.4)	-50
Cambodia	517 (308, 819)	138.5 (85.6, 222.2)	-61.7
Cameroon	3045 (1914, 4210)	295.1 (185.4, 411.3)	-25.5
Canada	34 (28, 42)	9.5 (7.6, 11.7)	130.8
Central African Republic	963 (583, 1480)	503.5 (300.1, 783.1)	-22.3
Chad	3892 (2525, 5588)	452.3 (298, 649.9)	-13.3
Chile	47 (38, 57)	23.7 (19.3, 28.7)	-45.9
China	1513 (1075, 2078)	14.1 (9.9, 19.5)	-79.3
Colombia	442 (321, 578)	65.4 (48.8, 85.4)	-29.5

Location	Number (95% UI)	MMR per 100,000 live births (95% UI)	Percentage change in the MMR (%)*
Comoros	33 (20, 53)	190 (112, 304.3)	-28.8
Congo	448 (264, 719)	347.9 (205.3, 549.9)	-19.2
Cook Islands	0 (0, 0)	3.9 (2.1, 6.6)	-24.8
Costa Rica	15 (12, 18)	27.4 (22.5, 33.3)	-8.9
Côte d'Ivoire	2610 (1550, 4131)	274.3 (158.9, 422.1)	-15.4
Croatia	1 (1, 1)	2.4 (1.8, 3)	-83.2
Cuba	46 (38, 57)	46.4 (37.9, 56.4)	-16
Cyprus	1 (0, 1)	3.7 (2.2, 6)	-63.8
Czechia	2 (2, 2)	1.8 (1.4, 2.3)	-85.9
Democratic People's Republic of Korea	173 (91, 304)	57.8 (31, 100.5)	-26.9
Democratic Republic of the Congo	11694 (7679, 16353)	409.2 (271.4, 573.4)	-0.6
Denmark	1 (1, 2)	2.1 (1.6, 2.7)	-67.4
Djibouti	126 (68, 210)	501.5 (260.8, 841.3)	-27.5
Dominica	1 (0, 1)	89.9 (51, 145.3)	164.2
Dominican Republic	207 (151, 276)	96.9 (69.8, 130.2)	6
Ecuador	263 (195, 351)	81.8 (59.5, 108.7)	-40.2
Egypt	876 (563, 1255)	33.6 (21.7, 48.3)	-77.7
El Salvador	49 (33, 68)	42.7 (29.4, 59.3)	-77.8
Equatorial Guinea	87 (40, 160)	231.6 (103.8, 431.4)	-61.2
Eritrea	832 (460, 1351)	425 (236.2, 690.1)	-51.1
Estonia	0 (0, 1)	3.2 (2.5, 3.8)	-90.8
Eswatini	38 (17, 71)	129.2 (57.9, 249.2)	12.8
Ethiopia	7642 (5086, 10471)	218.4 (148.5, 300.9)	-70.7
Fiji	16 (9, 28)	98.6 (54.4, 162.8)	-14.1
Finland	2 (1, 2)	3.9 (2.9, 5.1)	-30.6
France	34 (27, 43)	4.9 (3.9, 6.2)	-61.1
Gabon	81 (44, 136)	185.6 (98.4, 316.3)	-38.1
Gambia	318 (189, 493)	407.5 (244.4, 629.1)	-18.2
Georgia	9 (7, 11)	20.3 (16.3, 24.8)	-57.6
Germany	27 (24, 31)	3.5 (3, 4)	-67.2
Ghana	1708 (1184, 2486)	176.7 (122, 255.7)	-25.3
Greece	4 (3, 5)	4.8 (3.9, 5.6)	-33.1
Greenland	0 (0, 0)	14.5 (8.5, 22.9)	4.4
Grenada	1 (1, 1)	74.7 (57.4, 94)	111
Guam	1 (1, 2)	40.7 (26.7, 59.4)	50.1
Guatemala	546 (423, 688)	158.3 (122, 197.1)	-39.6
Guinea	2021 (1243, 3050)	407.5 (250.2, 610.1)	-38.6
Guinea-Bissau	161 (94, 270)	223.4 (132.3, 362.2)	-45.1
Guyana	25 (17, 36)	165.7 (109.6, 235.5)	102
Haiti	1460 (898, 2165)	423.1 (259.8, 623)	-7.2
Honduras	251 (121, 455)	114.2 (55.2, 204.8)	-51.6

Location	Number (95% UI)	MMR per 100,000 live births (95% UI)	Percentage change in the MMR (%)*
Hungary	4 (4, 6)	5.1 (4.1, 6.3)	-74.5
Iceland	0 (0, 0)	2 (1.7, 2.4)	-44.1
India	29007 (24349, 34980)	129.6 (108.6, 154.9)	-74.5
Indonesia	6366 (4782, 8881)	145 (107.8, 204.4)	-69.3
Iran (Islamic Republic of)	165 (112, 221)	16.1 (11, 21.6)	-64.6
Iraq	267 (156, 444)	28.6 (16.3, 48.5)	-57
Ireland	2 (1, 2)	3 (2.5, 3.5)	-46.6
Israel	5 (4, 6)	2.9 (2.4, 3.5)	-65.8
Italy	11 (9, 13)	2.9 (2.3, 3.4)	-67.3
Jamaica	24 (16, 36)	73.8 (48.4, 110.2)	127.3
Japan	42 (37, 51)	5.1 (4.4, 6)	-67.3
Jordan	58 (34, 90)	27.6 (15.7, 43.3)	-77.7
Kazakhstan	44 (34, 54)	10.2 (8, 12.9)	-79.8
Kenya	3459 (2314, 4998)	291.7 (193.8, 424.7)	-19.6
Kiribati	5 (3, 9)	181.2 (103.8, 296.5)	-13.5
Kuwait	4 (3, 5)	7 (5.6, 9)	30
Kyrgyzstan	46 (35, 57)	28.9 (22.1, 36.6)	-59.5
Lao People's Democratic Republic	272 (132, 502)	153.3 (76.2, 278.6)	-76.8
Latvia	1 (1, 1)	4.5 (3.6, 5.7)	-87.7
Lebanon	12 (7, 20)	15.3 (8.7, 25.1)	-60
Lesotho	141 (83, 229)	331.4 (193.5, 528.3)	23.6
Liberia	923 (577, 1381)	564.1 (348.3, 840.3)	-18
Libya	47 (26, 78)	59.2 (31.7, 99.7)	15.9
Lithuania	1 (1, 1)	3.4 (2.7, 4.3)	-85.2
Luxembourg	0 (0, 0)	4.8 (3.8, 5.9)	-54.6
Madagascar	2526 (1690, 3498)	288.3 (188.4, 399.7)	-15.6
Malawi	1360 (824, 2140)	236.6 (141, 371.7)	-27.1
Malaysia	312 (229, 412)	65.8 (48.1, 87)	-28.6
Maldives	4 (2, 5)	62.6 (40.6, 91.3)	-78.6
Mali	2891 (2089, 4010)	271.4 (197.2, 370.6)	-48.4
Malta	0 (0, 0)	8.1 (6.7, 9.8)	-35.2
Marshall Islands	1 (1, 3)	122.8 (65.2, 217.9)	54.4
Mauritania	524 (281, 859)	387.6 (208.7, 634.9)	-58.5
Mauritius	7 (6, 9)	57.9 (48.3, 69.1)	2.1
Mexico	858 (686, 1069)	46.2 (36.5, 57.7)	-38
Micronesia (Federated States of)	2 (1, 4)	133.1 (70.1, 224.9)	-18
Monaco	0 (0, 0)	9.1 (3.8, 16.9)	-2.3
Mongolia	31 (21, 45)	38.5 (25.5, 55.3)	-70.3
Montenegro	0 (0, 1)	6.2 (3.6, 10.1)	-43.1
Morocco	592 (319, 991)	91.7 (49.2, 157.5)	-74.6
Mozambique	2400 (1459, 3694)	216.7 (134.2, 327.3)	-34.3

Location	Number (95% UI)	MMR per 100,000 live births (95% UI)	Percentage change in the MMR (%)*
Myanmar	1611 (1078, 2247)	150 (99.7, 210.5)	-26.7
Namibia	65 (32, 123)	111.1 (54.3, 202.8)	-49.6
Nauru	0 (0, 1)	156.3 (84.4, 273.3)	-0.6
Nepal	1220 (679, 2107)	190 (106.6, 317.8)	-67.5
Netherlands	7 (6, 9)	4.1 (3.3, 4.9)	-57.4
New Zealand	3 (3, 4)	5.8 (5, 7.1)	-45.3
Nicaragua	59 (45, 76)	46.4 (35.6, 59.8)	-40.5
Niger	3943 (2450, 5804)	335.4 (209.2, 496.8)	-26
Nigeria	24904 (15771, 37461)	298.8 (192.2, 447.7)	-30.4
Niue	0 (0, 0)	112.6 (67.9, 176.7)	70.9
North Macedonia	1 (1, 2)	7 (4.2, 11.2)	-62.3
Northern Mariana Islands	1 (0, 1)	93.2 (53, 149.7)	35
Norway	1 (1, 1)	1.8 (1.4, 2.1)	-68.4
Oman	16 (9, 26)	19 (11, 31.7)	-42.1
Pakistan	11741 (6502, 19503)	189.9 (106, 319.7)	-38.6
Palau	1 (0, 1)	319 (180.7, 523.8)	39.9
Palestine	17 (10, 26)	14.3 (8.4, 22)	-44.2
Panama	48 (37, 60)	68.9 (53, 86.3)	0.9
Papua New Guinea	734 (478, 1054)	212.6 (139.2, 301.3)	-22
Paraguay	124 (84, 171)	96.8 (65.2, 136.4)	-14.1
Peru	613 (378, 904)	90.7 (56.2, 136.6)	-55.4
Philippines	1798 (1351, 2460)	82.3 (60.6, 113.1)	-13.1
Poland	6 (5, 7)	1.8 (1.4, 2.2)	-88.8
Portugal	6 (5, 7)	7 (5.8, 8)	-49.5
Puerto Rico	8 (6, 10)	41.3 (32.1, 53.1)	83.5
Qatar	4 (2, 7)	10.2 (5.6, 17.4)	-69.4
Republic of Korea	26 (18, 36)	9.7 (6.8, 13.3)	-55.2
Republic of Moldova	4 (3, 4)	12.5 (10.1, 15.3)	-75.5
Romania	21 (17, 25)	11.7 (9.4, 14.3)	-85
Russian Federation	150 (123, 184)	11.1 (9, 13.7)	-78.7
Rwanda	907 (584, 1289)	242.9 (157.2, 349.7)	-53.4
Saint Kitts and Nevis	1 (1, 1)	202.3 (151.3, 260.1)	-17.6
Saint Lucia	2 (1, 2)	92.9 (71.2, 119.4)	102.2
Saint Vincent and the Grenadines	1 (1, 1)	93.5 (73, 116.3)	93.4
Samoa	2 (1, 3)	26.9 (14.9, 45.7)	-34.3
San Marino	0 (0, 0)	3 (1.3, 5.5)	-40.8
Sao tome and Principe	4 (2, 8)	86.7 (44.5, 155.7)	-51
Saudi Arabia	414 (243, 660)	89.5 (52.8, 142.9)	-6.1
Senegal	1503 (1015, 2200)	313.5 (212.7, 450.3)	-24.6
Serbia	5 (3, 8)	8.4 (5.3, 12.7)	-61.4
Seychelles	1 (1, 1)	61.5 (39.9, 89.7)	6.3

Location	Number (95% UI)	MMR per 100,000 live births (95% UI)	Percentage change in the MMR (%)*
Sierra Leone	1376 (822, 2075)	454.3 (270.2, 679.6)	-15.7
Singapore	2 (1, 2)	2.8 (2.4, 3.4)	-74.2
Slovakia	3 (2, 4)	4.7 (3, 7.2)	-65.2
Slovenia	0 (0, 0)	1.4 (1, 1.8)	-89.5
Solomon Islands	65 (40, 96)	315 (195.8, 473.4)	21.1
Somalia	4417 (2746, 6493)	460 (292, 681)	-37.3
South Africa	1412 (1079, 1795)	143 (108.6, 181.2)	-34.5
South Sudan	1862 (1090, 2992)	484.4 (274.5, 782.4)	-6.9
Spain	13 (11, 15)	3.8 (3.2, 4.4)	-50.4
Sri Lanka	70 (43, 108)	23.5 (14.2, 36)	-70
Sudan	2295 (1145, 3906)	196.5 (98.8, 338.6)	-51.7
Suriname	12 (8, 18)	138.1 (83.7, 204.7)	48.3
Sweden	3 (3, 4)	2.8 (2.2, 3.5)	-39
Switzerland	2 (2, 3)	2.5 (2, 2.9)	-65.3
Syrian Arab Republic	66 (36, 114)	33.8 (18, 59.6)	-65.9
Taiwan (Province of China)	12 (10, 14)	7.8 (6.5, 8.9)	-45.7
Tajikistan	71 (46, 103)	24.9 (16.4, 36)	-60.5
Thailand	362 (224, 572)	63.2 (38.2, 100.5)	5.5
Timor-Leste	78 (47, 122)	189.9 (117.2, 297.1)	-69.9
Togo	527 (299, 850)	216.2 (121.7, 338.1)	-35.6
Tokelau	0 (0, 0)	115.8 (68.8, 181.8)	34.3
Tonga	3 (1, 4)	88.1 (50.2, 142.6)	-6
Trinidad and tobago	14 (10, 19)	93 (65.2, 127.8)	55.3
Tunisia	57 (29, 101)	34.3 (18.1, 58.3)	-53.5
Türkiye	278 (172, 443)	26.4 (16.2, 41.3)	-79.1
Turkmenistan	66 (47, 94)	59.8 (40.9, 86.3)	24.7
Tuvalu	0 (0, 0)	90.2 (49.2, 153.9)	-26.6
Uganda	2680 (1663, 3824)	168.2 (105.5, 238.1)	-34.5
Ukraine	38 (20, 61)	13.8 (7.4, 22.2)	-59.1
United Arab Emirates	18 (11, 29)	24.8 (14.5, 40.6)	-12.9
United Kingdom	30 (25, 34)	4.3 (3.6, 5)	-53.3
United Republic of Tanzania	6076 (4067, 8424)	319.4 (212.2, 445.5)	-17.7
United States of America	1079 (927, 1286)	29.6 (25.3, 35.3)	125.3
United States Virgin Islands	0 (0, 1)	42.5 (24.2, 66)	-12.3
Uruguay	10 (8, 13)	28.1 (23.1, 36.1)	-10.1
Uzbekistan	176 (131, 229)	22.2 (16.3, 29)	-44.1
Vanuatu	7 (4, 11)	79.9 (45.5, 132.5)	10.9
Venezuela (Bolivarian Republic of)	430 (302, 585)	104.2 (73.2, 141.2)	43.4
Viet Nam	263 (146, 441)	17 (9.4, 29.2)	-62.5
Yemen	1935 (1074, 3294)	195.5 (108.8, 329.3)	-54.1
Zambia	1106 (654, 1697)	182.3 (105.4, 279.8)	-35.3

**Location**	**Number (95% UI)**	**MMR per 100,000 live births (95% UI)**	**Percentage change in the MMR (%)***
Zimbabwe	1353 (795, 2137)	287.9 (167.8, 449.8)	48.2

MMR ─ maternal mortality ratio, UI ─ uncertainty intervals.

*Percentage change for each metric is in comparison to the corresponding point estimate in 1990.

Table S4. DALYs of maternal disorders in 2021, and the percentage change in the age ASR per 100,000 population compared to 1990, by country.

Location	Number (95% UI)	ASR per 100,000 population (95% UI)	Percentage change in the ASR (%)
Afghanistan	226906 (137846, 343835)	2349.2 (1427.1, 3559.7)	-49.1
Albania	215 (146, 302)	27.5 (18.7, 38.7)	-82.1
Algeria	37335 (23658, 54926)	262.2 (166.2, 385.8)	-74.5
American Samoa	57 (35, 87)	355.7 (217.9, 544.3)	-8.6
Andorra	3 (2, 5)	12.1 (7.9, 17.6)	-49.6
Angola	136662 (83490, 214333)	1320.6 (806.8, 2071.1)	-72.2
Antigua and Barbuda	30 (25, 35)	97.2 (83.7, 113.9)	-25.4
Argentina	17541 (13883, 22013)	117.9 (93.3, 147.9)	-58.3
Armenia	315 (251, 388)	34.4 (27.4, 42.3)	-77.3
Australia	1590 (1149, 2135)	20.8 (15, 27.9)	-37.2
Austria	436 (300, 580)	17.2 (11.9, 22.9)	-47
Azerbaijan	1662 (1201, 2236)	48.1 (34.8, 64.7)	-75.4
Bahamas	258 (196, 339)	187.3 (142.1, 245.7)	-15.6
Bahrain	406 (281, 573)	100.3 (69.4, 141.4)	-66.7
Bangladesh	270826 (186779, 371851)	468.7 (323.2, 643.5)	-88.6
Barbados	105 (82, 136)	115.2 (89.6, 149.5)	-30.4
Belarus	894 (663, 1174)	33 (24.5, 43.3)	-63.5
Belgium	657 (465, 879)	20.6 (14.6, 27.5)	-33.9
Belize	368 (308, 444)	241.9 (202.3, 291.4)	-12.2
Benin	75925 (48211, 107001)	1779.6 (1130.1, 2508.1)	-64.3
Bermuda	13 (11, 16)	74.1 (60.3, 90.4)	-31.5
Bhutan	1307 (734, 2133)	512.8 (287.9, 836.7)	-87.8
Bolivia (Plurinational State of)	29242 (20839, 41613)	745.3 (531.1, 1060.6)	-69
Bosnia and Herzegovina	158 (109, 223)	17 (11.7, 24)	-76.2
Botswana	2812 (1883, 3848)	334.1 (223.8, 457.3)	-67.8
Brazil	131131 (118699, 144599)	179.5 (162.5, 197.9)	-67.7
Brunei Darussalam	87 (59, 131)	57.3 (38.4, 86)	-75.1
Bulgaria	444 (350, 566)	24.2 (19.1, 30.8)	-70.5
Burkina Faso	127411 (88875, 182263)	1759.9 (1227.6, 2517.5)	-43
Burundi	83735 (53439, 118039)	2012.3 (1284.2, 2836.7)	-67.1
Cabo Verde	375 (233, 578)	199.5 (123.8, 307.4)	-79.3
Cambodia	30153 (18764, 47067)	527 (327.9, 822.6)	-81
Cameroon	191318 (123164, 261082)	1865.8 (1201.1, 2546.1)	-50
Canada	2833 (2289, 3437)	26.8 (21.7, 32.5)	20.8
Central African Republic	58944 (36772, 89077)	3248.7 (2026.7, 4909.4)	-46.1
Chad	242149 (159130, 345176)	4608.9 (3028.8, 6569.8)	-20.2
Chile	4072 (3356, 4874)	68.5 (56.5, 82)	-66.9
China	124642 (95533, 161794)	29.8 (22.8, 38.6)	-88.6
Colombia	29658 (22291, 37618)	181.2 (136.2, 229.8)	-64

Location	Number (95% UI)	ASR per 100,000 population (95% UI)	Percentage change in the ASR (%)
Comoros	2358 (1561, 3456)	944.3 (625.2, 1384)	-64.8
Congo	26980 (16499, 42180)	1457.8 (891.5, 2279.2)	-55.2
Cook Islands	1 (1, 2)	22.6 (15.2, 32.7)	-54.3
Costa Rica	1114 (930, 1345)	69 (57.6, 83.4)	-60.7
Côte d'Ivoire	159475 (96796, 249516)	1850.4 (1123.2, 2895.2)	-40.1
Croatia	151 (110, 201)	13.2 (9.6, 17.5)	-68.4
Cuba	3222 (2656, 3865)	97.9 (80.7, 117.4)	-46.9
Cyprus	78 (54, 108)	18 (12.5, 24.8)	-66.6
Czechia	467 (322, 678)	16 (11, 23.2)	-63.8
Democratic People's Republic of Korea	10893 (6087, 18668)	128.2 (71.6, 219.7)	-67
Democratic Republic of the Congo	704652 (462731, 975014)	2487.6 (1633.6, 3442.1)	-37.6
Denmark	253 (176, 336)	15.6 (10.8, 20.7)	-39.3
Djibouti	7953 (4438, 12893)	1980.2 (1105, 3210.3)	-58.3
Dominica	39 (23, 58)	180.9 (108.2, 272.3)	-17.3
Dominican Republic	13360 (10024, 17662)	368.4 (276.4, 487.1)	-36.7
Ecuador	17662 (13278, 22728)	295.5 (222.1, 380.2)	-62.8
Egypt	59626 (40947, 81520)	179.1 (123, 244.9)	-83.2
El Salvador	3302 (2304, 4473)	147.7 (103.1, 200.1)	-88.2
Equatorial Guinea	5410 (2579, 9771)	1153.7 (550.1, 2083.8)	-79.7
Eritrea	52414 (30353, 83269)	2457.9 (1423.4, 3904.8)	-67.6
Estonia	104 (70, 147)	29 (19.5, 41.1)	-74.4
Eswatini	2387 (1160, 4267)	599.9 (291.4, 1072.1)	-35.3
Ethiopia	488883 (340976, 655695)	1365.9 (952.7, 1832)	-80.8
Fiji	1017 (593, 1622)	344.9 (201.2, 549.9)	-34.2
Finland	271 (195, 359)	18.6 (13.4, 24.6)	-29
France	4420 (3345, 5703)	24 (18.1, 30.9)	-47.3
Gabon	5039 (2823, 8345)	795.6 (445.8, 1317.5)	-65.1
Gambia	18996 (11525, 29154)	2380.7 (1444.4, 3653.7)	-49.7
Georgia	621 (504, 743)	61.4 (49.8, 73.4)	-57.8
Germany	4984 (3541, 6800)	22.6 (16.1, 30.9)	-38
Ghana	109712 (78231, 154963)	943 (672.4, 1332)	-52.7
Greece	453 (350, 575)	16.1 (12.4, 20.4)	-29.1
Greenland	9 (6, 13)	53.7 (36.2, 78)	-32.9
Grenada	67 (53, 84)	204.5 (160.8, 255.5)	-16
Guam	71 (49, 98)	149.9 (105, 207.8)	-15.7
Guatemala	33646 (26133, 42097)	608.9 (472.9, 761.8)	-72.3
Guinea	125064 (78845, 186050)	2869.1 (1808.8, 4268.2)	-55.9
Guinea-Bissau	10386 (6306, 16885)	1520.9 (923.5, 2472.7)	-60.2
Guyana	1612 (1090, 2226)	629.3 (425.7, 868.9)	9.4
Haiti	86881 (53896, 127641)	1952.1 (1211, 2867.9)	-43
Honduras	15521 (7853, 27570)	435.2 (220.2, 773.1)	-76.7

Location	Number (95% UI)	ASR per 100,000 population (95% UI)	Percentage change in the ASR (%)
Hungary	515 (393, 645)	18.9 (14.5, 23.7)	-66.6
Iceland	18 (13, 24)	17.4 (12.4, 23.2)	-38
India	2002208 (1698270, 2388348)	421.7 (357.7, 503.1)	-85.7
Indonesia	382062 (292068, 527622)	404.1 (308.9, 558.1)	-81.1
Iran (Islamic Republic of)	14757 (11028, 18950)	51.2 (38.3, 65.7)	-82.4
Iraq	18212 (11684, 28300)	134.5 (86.3, 208.9)	-77.3
Ireland	254 (187, 330)	16.9 (12.4, 21.9)	-39.6
Israel	921 (680, 1220)	32.2 (23.8, 42.6)	-41.4
Italy	2004 (1459, 2566)	12.5 (9.1, 16)	-54.5
Jamaica	1584 (1106, 2270)	165.5 (115.5, 237.1)	-9
Japan	4807 (3742, 5989)	14.9 (11.6, 18.6)	-61.3
Jordan	3922 (2480, 5841)	98 (61.9, 145.9)	-89
Kazakhstan	3663 (2915, 4473)	60.4 (48.1, 73.8)	-74
Kenya	210238 (144175, 300258)	1224.6 (839.8, 1749)	-60.8
Kiribati	321 (188, 503)	784 (458.5, 1229.5)	-46.8
Kuwait	401 (305, 518)	23.2 (17.7, 30)	-50.8
Kyrgyzstan	3092 (2479, 3795)	139.4 (111.7, 171.1)	-68.2
Lao People's Democratic Republic	16953 (8422, 30812)	681.2 (338.5, 1238.2)	-87.7
Latvia	153 (110, 208)	30.2 (21.8, 41.2)	-75
Lebanon	977 (647, 1455)	53.3 (35.4, 79.5)	-77.7
Lesotho	8712 (5232, 13859)	1350.2 (810.9, 2147.8)	-24.8
Liberia	54338 (34201, 80170)	3017.7 (1899.4, 4452.3)	-55.9
Libya	2848 (1662, 4596)	115.3 (67.3, 186.2)	-67.6
Lithuania	207 (140, 293)	27.8 (18.9, 39.4)	-69.8
Luxembourg	33 (25, 41)	16.9 (13, 21.2)	-52.2
Madagascar	160612 (111299, 217817)	1686 (1168.3, 2286.4)	-47
Malawi	85160 (52432, 131922)	1287 (792.4, 1993.8)	-62.5
Malaysia	19733 (14808, 25549)	188.9 (141.8, 244.6)	-61.1
Maldives	247 (169, 340)	175 (119.6, 240.8)	-92.9
Mali	181090 (132866, 248475)	2482.9 (1821.7, 3406.8)	-56.1
Malta	36 (29, 45)	30.7 (24.5, 38.7)	-39.3
Marshall Islands	87 (49, 149)	456.3 (255.6, 784.2)	-18.6
Mauritania	31003 (17259, 49998)	2179.5 (1213.3, 3514.8)	-71.8
Mauritius	453 (385, 533)	114 (96.8, 134)	-48.9
Mexico	62844 (51469, 75913)	141.6 (116, 171)	-68.8
Micronesia (Federated States of)	148 (81, 246)	437.2 (239.3, 725.3)	-55.6
Monaco	3 (2, 4)	26.7 (16.4, 41.1)	-13.4
Mongolia	1981 (1387, 2812)	183 (128.2, 259.9)	-79
Montenegro	49 (34, 69)	26.8 (18.7, 37.7)	-43
Morocco	35556 (20229, 58811)	290.3 (165.1, 480.1)	-86.3
Mozambique	158008 (102410, 230916)	1562.1 (1012.5, 2282.9)	-50.6

**Location**	**Number (95% UI)**	**ASR per 100,000 population (95% UI)**	**Percentage change in the ASR (%)**
Myanmar	99395 (67701, 138051)	515.5 (351.1, 715.9)	-52.7
Namibia	4079 (2228, 7347)	483 (263.8, 870)	-69.7
Nauru	28 (16, 47)	762.4 (430.2, 1303.5)	-30.1
Nepal	76797 (43760, 129450)	681.4 (388.2, 1148.5)	-85.9
Netherlands	909 (694, 1172)	19.1 (14.6, 24.6)	-45.8
New Zealand	508 (384, 669)	33.3 (25.1, 43.8)	-48.9
Nicaragua	3936 (3114, 4940)	172.4 (136.4, 216.4)	-71.2
Niger	244255 (153498, 354621)	3338 (2097.7, 4846.2)	-36.1
Nigeria	1595916 (1060073, 2371579)	2076.4 (1379.2, 3085.6)	-49.5
Niue	2 (1, 2)	325.2 (201.1, 497.9)	16.8
North Macedonia	148 (102, 208)	22.4 (15.5, 31.4)	-71.7
Northern Mariana Islands	32 (19, 49)	213.1 (127.5, 328)	-34.6
Norway	267 (181, 377)	17.2 (11.7, 24.3)	-47.4
Oman	1126 (722, 1740)	90.5 (58, 139.8)	-74.1
Pakistan	742126 (424408, 1214810)	959.7 (548.8, 1570.9)	-62.5
Palau	35 (21, 56)	691.7 (404.3, 1112.1)	-17
Palestine	1313 (885, 1834)	77.7 (52.4, 108.5)	-70.8
Panama	3147 (2506, 3846)	229.8 (182.9, 280.7)	-33.6
Papua New Guinea	45689 (29967, 65057)	1374.1 (901.3, 1956.6)	-31
Paraguay	7997 (5550, 11034)	335.2 (232.6, 462.5)	-54.7
Peru	38552 (25266, 55474)	321.8 (210.9, 463)	-72.5
Philippines	116351 (88741, 154142)	310.8 (237.1, 411.8)	-49.7
Poland	1410 (996, 1901)	12.8 (9.1, 17.3)	-78.8
Portugal	579 (457, 708)	19.4 (15.3, 23.7)	-51.8
Puerto Rico	552 (443, 685)	57.6 (46.3, 71.6)	-40.7
Qatar	337 (218, 499)	51.4 (33.2, 76)	-80.9
Republic of Korea	1893 (1421, 2486)	12.7 (9.5, 16.6)	-79.5
Republic of Moldova	378 (299, 475)	34.7 (27.4, 43.5)	-82.5
Romania	1991 (1572, 2482)	37.1 (29.3, 46.2)	-82
Russian Federation	18688 (14612, 24036)	43.5 (34, 56)	-71.2
Rwanda	55623 (36892, 77499)	1230.4 (816.1, 1714.3)	-73.6
Saint Kitts and Nevis	73 (55, 93)	373.6 (282.2, 476.2)	-66.2
Saint Lucia	100 (80, 126)	173.4 (138.4, 218.8)	-33.4
Saint Vincent and the Grenadines	80 (65, 98)	223.9 (183, 273.8)	-13.4
Samoa	119 (74, 190)	185.2 (114.5, 295.9)	-38.6
San Marino	1 (1, 2)	13.1 (8.6, 18.9)	-31.7
Sao tome and Principe	267 (149, 454)	364.8 (203.4, 621.4)	-74.9
Saudi Arabia	24272 (14903, 37527)	201.5 (123.7, 311.5)	-71.9
Senegal	90643 (61816, 131715)	1778.7 (1213, 2584.7)	-53.3
Serbia	546 (396, 727)	21.3 (15.5, 28.4)	-71.8
Seychelles	60 (42, 85)	192.2 (132, 270)	-28.3

Location	Number (95% UI)	ASR per 100,000 population (95% UI)	Percentage change in the ASR (%)
Sierra Leone	86106 (53173, 127964)	2951 (1822.3, 4385.6)	-45.3
Singapore	186 (140, 240)	10.5 (7.9, 13.6)	-74.6
Slovakia	325 (235, 446)	20.6 (14.9, 28.3)	-61.1
Slovenia	85 (56, 124)	15.7 (10.3, 22.7)	-61.2
Solomon Islands	3936 (2500, 5757)	1778.3 (1129.6, 2601)	-21.1
Somalia	270472 (173008, 388675)	4239.5 (2711.8, 6092.2)	-43.3
South Africa	90326 (70542, 111834)	466 (363.9, 576.9)	-60.8
South Sudan	114963 (70096, 180964)	3684.7 (2246.7, 5800.2)	-24.7
Spain	2174 (1591, 2842)	16.9 (12.3, 22)	-41.9
Sri Lanka	5277 (3631, 7639)	72.9 (50.1, 105.5)	-77.7
Sudan	143328 (76590, 235811)	986.9 (527.4, 1623.7)	-74.6
Suriname	790 (509, 1147)	420.1 (270.4, 609.6)	-12
Sweden	466 (341, 602)	16.5 (12.1, 21.3)	-40.5
Switzerland	443 (309, 598)	17.7 (12.4, 24)	-39.8
Syrian Arab Republic	4434 (2583, 7211)	86.8 (50.6, 141.2)	-88.9
Taiwan (Province of China)	1104 (904, 1333)	15.8 (12.9, 19)	-69.6
Tajikistan	4859 (3320, 6765)	149.6 (102.2, 208.2)	-72.1
Thailand	21708 (13744, 32979)	103.4 (65.5, 157.1)	-48.4
Timor-Leste	4655 (2857, 7194)	1039.6 (638.1, 1606.7)	-82.2
Togo	32191 (19057, 50554)	1154.5 (683.5, 1813.1)	-60.4
Tokelau	1 (1, 2)	312.1 (191.3, 477)	-23.5
Tonga	161 (96, 252)	485.4 (288.4, 758.2)	-23
Trinidad and tobago	870 (635, 1176)	203.2 (148.4, 274.7)	-18.2
Tunisia	3786 (2135, 6407)	98.1 (55.3, 166)	-75.4
Türkiye	19864 (13444, 29255)	73.3 (49.6, 108)	-88.7
Turkmenistan	4163 (2991, 5861)	258.5 (185.7, 363.8)	-23.1
Tuvalu	16 (9, 26)	421.9 (247.5, 703.9)	-54.5
Uganda	188441 (129675, 255744)	1362.8 (937.8, 1849.5)	-57.2
Ukraine	4250 (2782, 5860)	33 (21.6, 45.5)	-68.8
United Arab Emirates	1289 (847, 1902)	62.3 (41, 92)	-72.7
United Kingdom	4205 (3171, 5467)	21.3 (16.1, 27.7)	-46.4
United Republic of Tanzania	383140 (261943, 522872)	1969.5 (1346.5, 2687.8)	-47.7
United States of America	77525 (66850, 91130)	80.2 (69.2, 94.3)	30
United States Virgin Islands	23 (15, 34)	101 (64.8, 147.6)	-53.2
Uruguay	814 (666, 977)	77.6 (63.6, 93.2)	-41.3
Uzbekistan	12500 (9682, 15954)	112.1 (86.8, 143)	-61.8
Vanuatu	445 (264, 704)	440.9 (261.9, 698.2)	-30.2
Venezuela (Bolivarian Republic of)	27771 (20067, 37009)	316.5 (228.7, 421.8)	-24.7
Viet Nam	20071 (13011, 30526)	62 (40.2, 94.4)	-77.1
Yemen	129297 (77027, 210915)	1180.5 (703.3, 1925.7)	-75.9
Zambia	74367 (46930, 110319)	1156.2 (729.7, 1715.2)	-60.3

Location	Number (95% UI)	ASR per 100,000 population (95% UI)	Percentage change in the ASR (%)
Zimbabwe	83999 (51677, 130736)	1591.4 (979, 2476.8)	5.4

DALYs ─ disability-adjusted life years, ASR ─ age-standardised rate, UI ─ uncertainty intervals.

*Percentage change for each metric is in comparison to the corresponding point estimate in 1990.

Table S5. Global forecast of incidence cases and ASR for maternal disorders from 2022 to 2036 predicted by the BAPC model.

Year	Predicted 10-54 ages number (95% CI)	Predicted ASR of incidence per 100,000 population (95% CI)
1990	114968054.3 (114938815.1, 114997293.5)	6748.1 (6746.9, 6749.3)
1991	113793100.9 (113764012.7, 113822189.1)	6572.4 (6571.2, 6573.5)
1992	112782314.8 (112753349.5, 112811280.1)	6411.5 (6410.3, 6412.6)
1993	112017174.6 (111988293.8, 112046055.4)	6267.3 (6266.2, 6268.4)
1994	111634489.5 (111605633.2, 111663345.9)	6146.3 (6145.2, 6147.4)
1995	111750458.8 (111721555.4, 111779362.2)	6053.3 (6052.3, 6054.4)
1996	112239049.0 (112210041.5, 112268056.5)	5978.9 (5977.8, 5979.9)
1997	112819738.6 (112790611.2, 112848866.1)	5908.6 (5907.6, 5909.7)
1998	113463588.7 (113434334.6, 113492842.9)	5841.8 (5840.8, 5842.9)
1999	114165468.9 (114136084.9, 114194852.9)	5779.6 (5778.6, 5780.7)
2000	114927341.3 (114897825.0, 114956857.5)	5722.2 (5721.2, 5723.3)
2001	115528681.6 (115499061.6, 115558301.7)	5662.8 (5661.8, 5663.8)
2002	115823232.8 (115793559.7, 115852905.9)	5597.4 (5596.4, 5598.4)
2003	116065509.0 (116035794.6, 116095223.3)	5532.2 (5531.2, 5533.2)
2004	116254797.0 (116225059.7, 116284534.3)	5470.9 (5469.9, 5471.8)
2005	116635068.2 (116605285.0, 116664851.4)	5417.7 (5416.7, 5418.7)
2006	116786184.8 (116756393.6, 116815975.9)	5360.8 (5359.8, 5361.7)
2007	116610335.5 (116580576.0, 116640095.1)	5291.6 (5290.6, 5292.5)
2008	116319814.9 (116290099.4, 116349530.4)	5220.3 (5219.4, 5221.2)
2009	116168450.9 (116138759.0, 116198142.7)	5158.6 (5157.7, 5159.5)
2010	116417151.5 (116387427.3, 116446875.6)	5119.4 (5118.4, 5120.3)
2011	116989640.8 (116959835.5, 117019446.1)	5097.0 (5096.1, 5097.9)
2012	117577744.4 (117547848.0, 117607640.8)	5075.1 (5074.2, 5076.0)
2013	118115306.6 (118085317.6, 118145295.7)	5050.7 (5049.8, 5051.6)
2014	118477095.9 (118447030.5, 118507161.3)	5019.7 (5018.8, 5020.6)
2015	118539842.9 (118509735.8, 118569949.9)	4977.7 (4976.8, 4978.6)
2016	118070054.3 (118039972.3, 118100136.2)	4915.5 (4914.6, 4916.3)
2017	117083963.9 (117053973.7, 117113954.1)	4834.1 (4833.2, 4835.0)
2018	115866142.8 (115836275.9, 115896009.7)	4746.5 (4745.6, 4747.4)
2019	114733262.1 (114703508.9, 114763015.3)	4664.7 (4663.8, 4665.5)
2020	113620969.8 (113591330.9, 113650608.6)	4586.3 (4585.4, 4587.1)
2021	113179912.1 (113150304.2, 113209520.1)	4536.8 (4535.9, 4537.6)
2022	112098091.5 (105490484.4, 118705698.5)	4404.6 (4145.0, 4664.2)
2023	110638466.3 (103141357.6, 118135575.1)	4318.6 (4026.0, 4611.3)
2024	109179256.8 (100476539.9, 117881973.7)	4234.7 (3897.2, 4572.3)
2025	107740950.3 (97568773.1, 117913127.6)	4153.4 (3761.3, 4545.6)
2026	106334883.7 (94476952.5, 118192815.0)	4074.9 (3620.5, 4529.3)
2027	104974981.2 (91252291.2, 118697671.2)	3998.8 (3476.1, 4521.6)
2028	103595942.7 (87875873.8, 119316011.7)	3924.6 (3329.1, 4520.2)

**Year**	**Predicted 10-54 ages number (95% CI)**	**Predicted ASR of incidence per 100,000 population (95% CI)**
2029	102230428.5 (84400585.5, 120060271.5)	3852.3 (3180.4, 4524.2)
2030	100887236.5 (80846749.3, 120927723.8)	3782.2 (3030.9, 4533.5)
2031	99570603.6 (77223794.4, 121917412.9)	3714.5 (2880.9, 4548.2)
2032	98276183.2 (73532851.6, 123019514.8)	3649.1 (2730.3, 4567.8)
2033	96988481.6 (69773272.2, 124203691.1)	3585.2 (2579.2, 4591.2)
2034	95700777.3 (65947713.5, 125453841.0)	3522.8 (2427.6, 4618.0)
2035	94410117.2 (62058827.9, 126761406.5)	3462.1 (2275.7, 4648.4)
2036	93114951.2 (58108881.5, 128121020.8)	3403.2 (2123.8, 4682.6)

ASR — age-standardised rate, BAPC — Bayesian age-period-cohort, CI — confidence interval.

Table S6. Global forecast of deaths cases and ASR of MMR from 2022 to 2036 predicted by the BAPC model.

**Year**	**Predicted 10-54 ages number (95% CI)**	**Predicted ASR of MMR per 100,000 live births (95% CI)**
1990	375885.5 (374108.5, 377662.5)	285.2 (284.2, 286.2)
1991	368326.2 (366568.9, 370083.4)	280.6 (279.6, 281.5)
1992	365353.3 (363606.2, 367100.4)	279.0 (278.0, 280.0)
1993	359916.0 (358186.1, 361645.9)	275.1 (274.1, 276.1)
1994	358154.0 (356431.3, 359876.8)	274.3 (273.3, 275.3)
1995	355063.5 (353352.1, 356774.9)	272.5 (271.5, 273.5)
1996	350643.8 (348947.7, 352339.9)	269.7 (268.8, 270.7)
1997	347067.6 (345384.1, 348751.1)	267.7 (266.7, 268.6)
1998	344385.1 (342711.4, 346058.8)	266.1 (265.1, 267.0)
1999	341017.0 (339355.4, 342678.7)	263.4 (262.4, 264.3)
2000	337399.5 (335748.8, 339050.2)	259.9 (259.0, 260.8)
2001	329587.5 (327956.2, 331218.9)	252.9 (252.0, 253.8)
2002	323396.2 (321780.6, 325011.9)	247.2 (246.3, 248.1)
2003	316699.6 (315101.8, 318297.4)	240.9 (240.0, 241.8)
2004	310990.5 (309409.9, 312571.2)	234.9 (234.0, 235.7)
2005	304681.6 (303120.9, 306242.2)	227.8 (227.0, 228.6)
2006	296372.0 (294836.7, 297907.4)	219.0 (218.2, 219.8)
2007	285944.0 (284439.2, 287448.8)	208.9 (208.1, 209.7)
2008	278505.2 (277021.1, 279989.2)	201.9 (201.1, 202.6)
2009	273258.0 (271787.1, 274729.0)	197.4 (196.6, 198.1)
2010	264699.4 (263251.0, 266147.9)	192.1 (191.4, 192.9)
2011	254930.2 (253510.3, 256350.1)	184.8 (184.0, 185.5)
2012	248322.2 (246922.7, 249721.7)	177.9 (177.2, 178.7)
2013	243163.4 (241779.7, 244547.1)	173.4 (172.7, 174.1)
2014	233308.8 (231955.4, 234662.3)	165.8 (165.1, 166.5)
2015	226217.1 (224887.1, 227547.1)	159.5 (158.9, 160.2)
2016	220979.2 (219667.6, 222290.9)	155.7 (155.0, 156.3)
2017	216047.8 (214753.8, 217341.9)	154.2 (153.6, 154.9)
2018	207531.4 (206265.7, 208797.2)	151.0 (150.4, 151.7)
2019	200548.0 (199306.1, 201789.9)	149.3 (148.7, 150.0)
2020	195207.1 (193983.7, 196430.5)	148.7 (148.1, 149.4)
2021	191136.2 (189926.2, 192346.3)	147.7 (147.1, 148.4)
2022	194976.9 (182648.0, 207305.7)	147.4 (138.1, 156.7)
2023	193442.6 (178538.9, 208346.3)	145.9 (134.7, 157.1)
2024	191914.2 (173617.0, 210211.3)	144.4 (130.7, 158.2)
2025	190439.6 (168099.2, 212779.9)	143.0 (126.2, 159.8)
2026	189058.2 (162148.7, 215967.7)	141.6 (121.5, 161.8)
2027	187815.8 (155890.3, 219741.2)	140.3 (116.5, 164.1)
2028	186669.7 (149355.5, 223983.8)	139.0 (111.2, 166.8)

Year	Predicted 10-54 ages number (95% CI)	Predicted ASR of MMR per 100,000 live births (95% CI)
2029	185556.5 (142536.8, 228576.1)	137.7 (105.8, 169.7)
2030	184478.9 (135465.8, 233492.1)	136.5 (100.2, 172.7)
2031	183433.4 (128158.0, 238708.9)	135.2 (94.5, 176.0)
2032	182435.7 (120638.4, 244233.0)	134.0 (88.6, 179.3)
2033	181416.0 (112882.0, 249950.0)	132.7 (82.6, 182.8)
2034	180381.6 (104912.1, 255851.0)	131.4 (76.4, 186.4)
2035	179304.4 (96726.2, 261882.6)	130.1 (70.2, 190.0)
2036	178151.8 (88320.8, 267982.9)	128.8 (63.8, 193.7)

ASR — age standardized rate, MMR — maternal mortality ratio, BAPC — Bayesian age-period-cohort, CI — confidence interval.
